# scGEN: Leveraging an Adaptive Weighting Strategy for Challenging Cell Clustering in Single‐Cell RNA‐seq

**DOI:** 10.1002/advs.77746

**Published:** 2026-10-11

**Authors:** Fang Huang, Dayu Hu, Yaqi Dai, Aoyi Geng, Renxiang Guan, Hao Quan, Brett A. McGregor, Zhihan Wang, Nadeem Khan, Hongsheng Zhang, Jinghua Zhang, Mingyang Xia, Xiaoqiong Gu, Di Che, Junguk Hur, Xiaoyu Cui, Kai Guo

**Affiliations:** ^1^ Department of Neurobiology School of Basic Medical Sciences Chongqing Medical University Chongqing People's Republic of China; ^2^ Key Laboratory of Major Brain Disease and Aging Research (Ministry of Education) Chongqing Medical University Chongqing People's Republic of China; ^3^ Department of Biomedical Sciences School of Medicine and Health Sciences University of North Dakota Grand Forks North Dakota USA; ^4^ College of Medicine and Biological Information Engineering Northeastern University Shenyang People's Republic of China; ^5^ Department of Human Anatomy School of Basic Medical Sciences Chengdu Medical College Chengdu People's Republic of China; ^6^ Department of Oral Biology College of Dentistry University of Florida Gainesville Florida USA; ^7^ College of Computer Science and Software Engineering Hohai University Nanjing People's Republic of China; ^8^ Institute of Pediatrics Guangzhou Women and Children's Medical Center Guangzhou Medical University Guangzhou People's Republic of China; ^9^ Key Laboratory of Pediatric Rare Diseases of Guangdong Higher Education Institutes Guangzhou Medical University Guangzhou People's Republic of China; ^10^ Department of Clinical Biological Resource Bank Guangzhou Women and Children's Medical Center Guangzhou Medical University Guangzhou People's Republic of China; ^11^ Department of Molecular Medical Center Guangzhou Women and Children's Medical Center Guangzhou Medical University Guangzhou People's Republic of China; ^12^ Department of Neurology University of Michigan Ann Arbor Michigan USA; ^13^ School of Medicine and Warshel Institute For Computational Biology The Chinese University of Hong Kong Shenzhen Guangdong People's Republic of China

**Keywords:** graph learning, hard‐sample learning, single‐cell clustering, zero‐inflated negative binomial loss (ZINB)

## Abstract

Recent advancements in single‐cell RNA sequencing (scRNA‐seq) have greatly enhanced our ability to dissect cellular heterogeneity. However, unsupervised clustering often struggles to identify transitional or developmental boundary cells, as existing methods rely on highly variable genes without considering expression levels, thereby overlooking subtle but crucial signals. To address this challenge, we developed a single‐cell gene‐aware embedded network (scGEN), which captures complex intercellular relationships. scGEN employs adaptive feature weighting and iterative fine‐tuning to prioritize ambiguous or transitional cells with overlapping transcriptional profiles. Evaluation across eight distinct scRNA‐seq datasets demonstrated that scGEN consistently outperformed nine leading clustering approaches. Additionally, scGEN generated refined assignments for approximately 10% of cells with ambiguous transcriptional profiles and revealed biologically meaningful differences, providing a more detailed view of cellular heterogeneity in the human fetal pituitary. Cell–cell communication analysis identified developmental signaling networks associated with the refined annotations, including a putative Pre.Gonado‐like population detected at 7 weeks with prominent involvement in BMP, NOTCH, and extracellular matrix remodeling pathways. Furthermore, histological analysis of 6–8‐week embryos provided orthogonal tissue‐level support for early gonadotrope‐lineage states and SOX2–GATA2–NR5A1 bridging populations. Together, these results highlight scGEN's ability to enhance cell‐type assignments and detect subtle biological differences.

## Graphical abstract

scGEN combines attribute–structure encoding, adaptive hard‐sample weighting, and regularized zero‐inflated negative binomial loss to resolve ambiguous single‐cell states. In the developing human pituitary, refined annotations reveal Pre.Gonado cells at 7 weeks and previously obscured developmental communication networks. Independent SOX2/GATA2/NR5A1 histology supports a progenitor‐to‐gonadotrope trajectory, linking improved clustering to early endocrine specification.

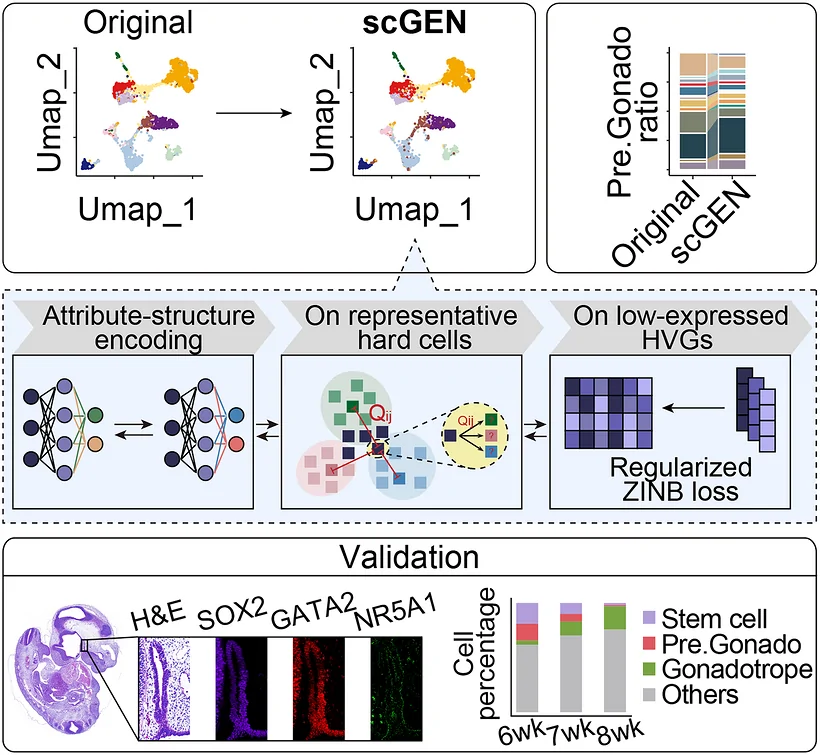

## 1 Introduction

Single‐cell sequencing techniques have broken through the limitations of bulk tissue analysis, revealing an unprecedented degree of cellular variation within seemingly homogeneous populations [1, 2]. Advances in single‐cell technologies allow biologists to explore cellular heterogeneity, enabling the discovery of novel cell types, an understanding of intricate differentiation and developmental mechanisms, and enhanced insight into human diseases [3, 4, 5, 6]. Recent advances have notably improved the availability and usefulness of single‐cell sequencing, enabling the analysis of tens of millions of cells in a single experiment. One of the main challenges in single‐cell analysis is accurately classifying cells into distinct types or states [7, 8, 9], especially for cells located in intermediate or mixed‐lineage regions, where identity is less well‐defined. This classification is essential for downstream analysis because of the challenges posed by the complexities and variabilities of cellular states, technological noise, and the sheer amount of high‐dimensional data generated.

Single‐cell clustering is an effective method for assigning distinct labels to cells without prior assumptions. However, single‐cell data pose formidable computational challenges for identifying and classifying cell types due to their complex properties [10]. Traditional clustering techniques, such as k‐means, do not effectively account for the complex, non‐linear relationships inherent in single‐cell data [11]. Deep clustering algorithms, based on deep learning, have been gradually incorporated into single‐cell analysis [12, 13, 14]. These algorithms extract more informative and discriminative feature representations directly from raw data, exhibiting better capacity for handling noise and batch effects than traditional methods [15, 16].

Deep clustering applications can be classified into several categories: (a) Deep embedding clustering methods, such as Deep Embedded Clustering (DEC) [17], extract low‐dimensional representations to enhance clustering performance. (b) Deep graph‐based clustering methods, such as Deep Graph Infomax (DGI) [18], use graph structures to model complex relationships among data points in the learned feature space, thereby facilitating clustering. (c) Deep fusion clustering methods [19], such as the Structure and Attribute Information Fusion (SAIF) module, integrate representations from autoencoders and graph autoencoders for comprehensive feature learning. (d) Variational Autoencoder (VAE)‐based clustering methods, including Variational Deep Embedding (VaDE) [20], utilize VAEs' generative features to gain latent representations for clustering. (e) Adversarial deep clustering methods, such as ClusterGAN [21], facilitate clustering using adversarial training to produce more distinct features. (f) Self‐supervised deep clustering methods, such as Invariant Information Clustering (IIC) [22], use pretext tasks to gather essential representations to cluster without labeled data. (g) Deep subspace clustering methods, such as DSC‐Nets [23], extend conventional subspace clustering approaches inside deep learning frameworks. However, many existing approaches optimize global cluster separation and treat samples uniformly during training, limiting their ability to resolve “hard samples” such as cells in transitional states, which often carry critical developmental information. As a result, transitional and boundary cell populations, often representing key developmental or lineage commitment stages, remain incompletely characterized in many single‐cell studies.

Hard‐sample learning effectively addresses these challenges by focusing on informative hard samples within datasets. The concept of hard‐sample learning originates from the observation that not all training examples contribute equally to model learning [24, 25]. While some samples are easily classified, others pose greater challenges and provide more valuable information for refining the model's decision boundaries. Approaches like Online Hard Example Mining (OHEM) [25], which selects challenging samples based on loss values, and other techniques for prioritizing hard cell samples by down‐weighting other cells [26], may be helpful in single‐cell data analysis due to the inherent heterogeneity and transitions between cellular states. However, these approaches have yet to be fully integrated into single‐cell clustering workflows.

To address these challenges, we have developed a gene‐aware embedding network (scGEN) designed for the complexity of single‐cell data clustering. scGEN focuses on hard samples, leading to improved clustering accuracy and enhanced biological interpretability. By integrating structure‐guided hard‐sample mining and adaptive weighting gene‐aware clustering into a unified framework, scGEN combines both expression profiling (attribute) and cell similarity (structural) information to prioritize transitional or boundary cell states, which are crucial for understanding cellular heterogeneity and developmental trajectories. This integration is achieved through dual encoders that extract gene expression features and intercellular structural relationships separately, which are then integrated into a global representation optimized through a structure‐guided contrastive loss. Additionally, scGEN employs a regularized zero‐inflated negative binomial (ZINB) model to characterize the probabilistic structure of the data and introduces a novel weighting strategy to enhance the detection of subtle yet essential features. Extensive benchmarking across diverse datasets highlights scGEN's capability to reveal hidden patterns in single‐cell data and addresses existing clustering methods’ limitations, offering a powerful tool for advancing our understanding of cellular diversity. Unlike general clustering frameworks, scGEN is designed to explicitly prioritize these hard‐to‐classify samples using a novel structure‐guided hard‐sample learning strategy combined with a gene‐aware regularized ZINB loss, which we demonstrate leads to consistent performance gains across diverse biological systems and enables meaningful biological reinterpretation of ambiguous cell states.

## 2 Results

### 2.1 scGEN Outperforms Other Methods in Single‐Cell Clustering Across Diverse Datasets

The computational architecture for scGEN is illustrated in Figure 1 (Experimental Section). Briefly, it contains three core components: (1) construction of a cell–cell similarity graph followed by initial clustering to identify challenging samples characterized by ambiguous cluster assignments, (2) parallel network encoding, where both attribute and structure information are processed through dual semantic networks to generate joint representations, and (3) implementation of a regularized ZINB loss framework that mitigates technical noise in sparsely expressed highly variable genes (HVGs) while preserving biologically meaningful signals through iterative refinement of reliable sample selection.

**FIGURE 1:**
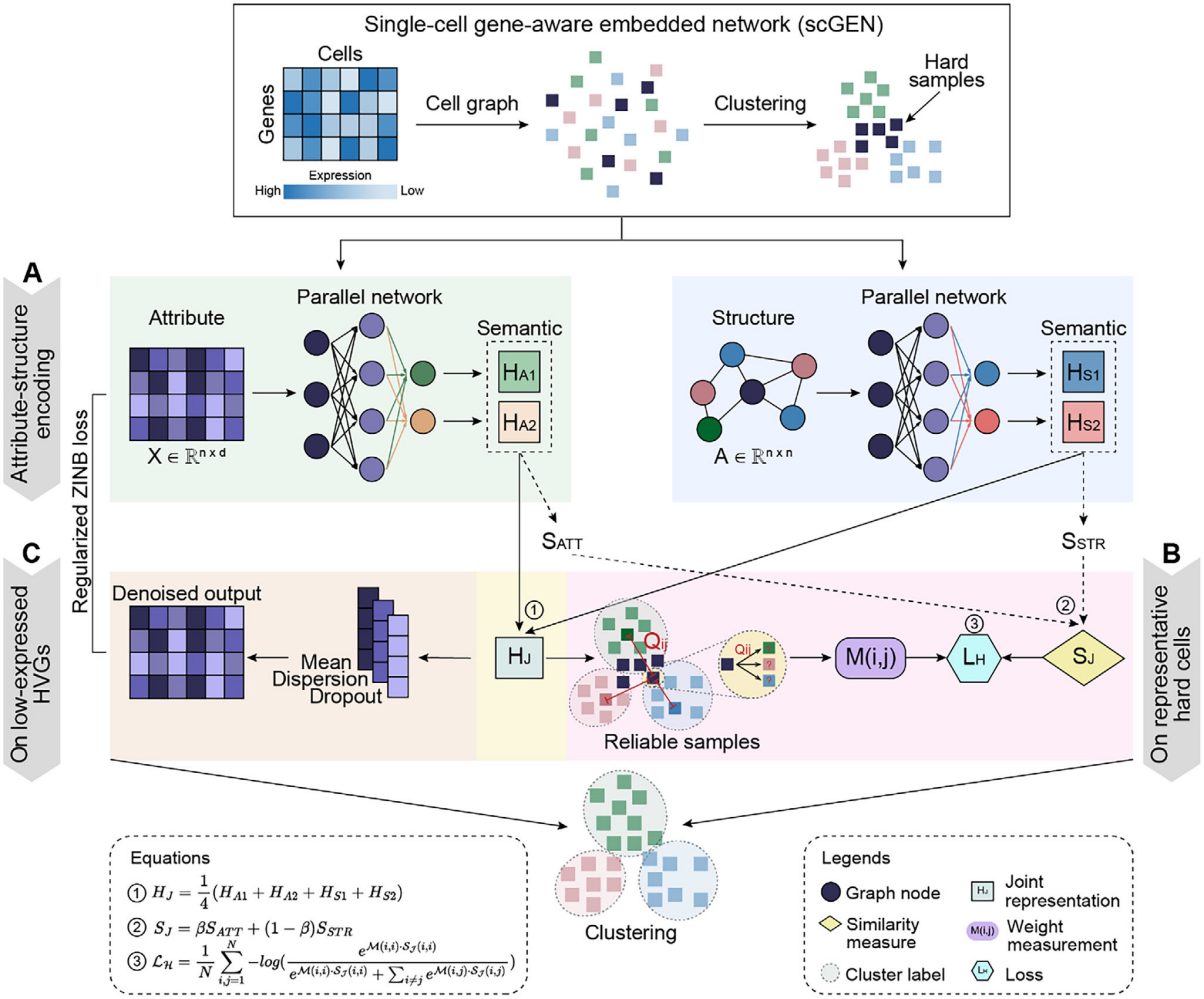
The framework of scGEN comprises three parts. (A) Joint attribute‐structure similarity is simultaneously learned by an attribute and structure autoencoder. (B) The constructed joint similarity guides the network to focus on representative hard samples. (C) The regularized ZINB loss module.

We then benchmarked scGEN's performance against nine state‐of‐the‐art clustering methods, including six deep learning‐based methods (AttentionAE [27], GraphSCC [28], scDEFR [29], scDeepCluster [30], scDCCA [12], and scTPC [31]) and three classical clustering methods (k‐means, Spectral Clustering, and SSNMDI [32]) using eight publicly available scRNA‐seq datasets with well‐defined cluster labels across human and mouse tissues (Figure 2; Experimental Section). Figure 2 and Table S1 provide qualitative and quantitative comparisons, demonstrating that scGEN consistently outperformed other approaches across various evaluation metrics. For instance, in the Hrvatin B1 dataset, scGEN achieved an accuracy (ACC) of 96.41%, and in the Scala dataset, it achieved a similarly high clustering accuracy (Figure 2A). Moreover, scGEN consistently exhibited the highest ACC scores across all datasets, indicating its robust ability to detect cell clusters that accurately mirrored the ground truth. Notably, in the Scala dataset, scGEN achieved an Adjusted Rand Index (ARI) [33] of 58.59%, considerably surpassing alternative approaches. These improvements in ARI and normalized mutual information (NMI) [34] reflect scGEN's strength in preserving biologically coherent cell populations, thereby facilitating more accurate downstream interpretations.

**FIGURE 2:**
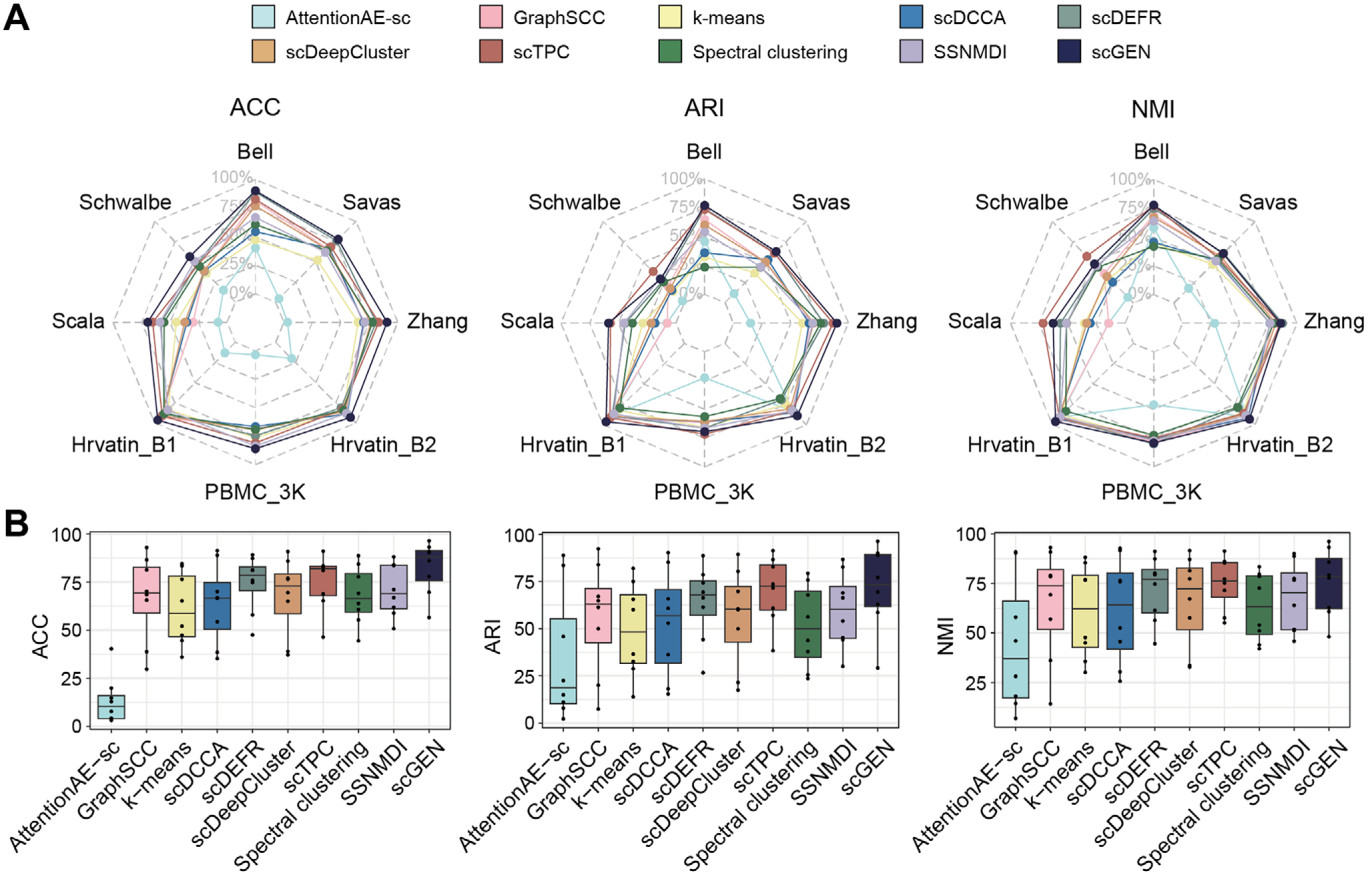
Qualitative clustering performance comparison of scGEN and baseline methods across multiple scRNA‐seq datasets. (A) Performance comparison of AttentionAE‐sc, GraphSCC, K‐means, scDCCA, scDEFR, scDeepCluster, scTPC, Spectral Clustering, SSNMDI, and scGEN on eight scRNA‐seq datasets, measured by ACC, NMI, and ARI. (B) Box plots illustrating the distribution of performance metrics (ACC, NMI, and ARI) across all datasets for each method.

To further validate scGEN's performance against widely used classical methods, we conducted additional comparisons with Seurat (version 5) and Scanpy clustering pipelines across multiple resolution parameters (Tables S3 and S4. scGEN consistently outperformed both classical methods across the majority of datasets. Particularly notable improvements were observed in challenging datasets such as the Zhang dataset (human fetal pituitary), where scGEN achieved 90.03% ARI compared to Seurat's best performance of 80.99% and Scanpy's best of 83.11%. These results demonstrate the advantages of scGEN over conventional clustering approaches, especially for datasets containing ambiguous or transitional cell populations.

In addition, scGEN demonstrated its robust performance in capturing the underlying biological structure, as evidenced by the ARI and NMI scores. scGEN achieved the highest ARI in six datasets and ranked second in the remaining two. scGEN also achieved the highest NMI in three datasets and ranked second in the remaining five, with a particularly high NMI score of 96.22% in the Hrvatin B1 dataset. Moreover, our results further indicated scGEN's enhanced performance stability across all datasets (Figure 2B), showing consistently elevated median values and lower variance in comparison with other approaches. Overall, these findings underscore scGEN's robust and consistent performance, achieving superior clustering accuracy and stability across a range of diverse single‐cell RNA‐seq datasets.

### 2.2 Parameter Sensitivity and Robustness of scGEN in Single‐Cell Clustering

scGEN utilizes two hyperparameters: α and γ, where α balances the contributions of the two loss components, and γ adjusts the attention weight assigned to hard samples in the learning process. To carefully investigate the influence of these parameters and assess the robustness and generalizability of our clustering algorithm, we conducted extensive parameter sensitivity analyses across the eight single‐cell datasets, with the parameter α varying from 0.01 to 100 and γ from 1 to 5 to determine the optimal parameter space. Figure 3 displays the ACC performance landscapes across multiple parameter combinations across all datasets. The model achieved maximum ACC performance at α = 1 and γ = 1 across most datasets.

**FIGURE 3:**
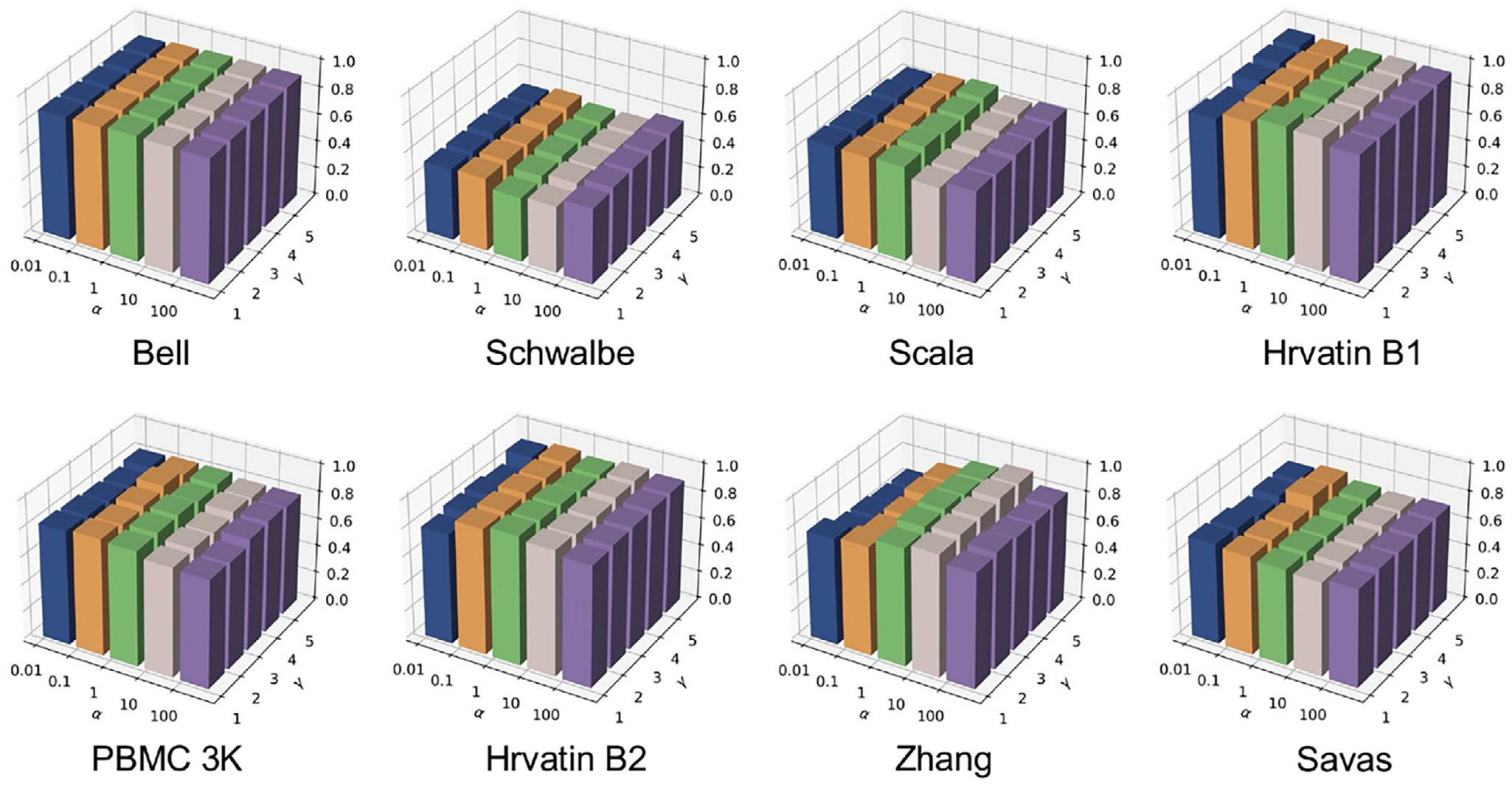
Parameter sensitivity analysis of scGEN across eight scRNA‐seq datasets. The three‐dimensional plots illustrate the impact of parameters α (ranging from 0.01 to 100) and γ (ranging from 1 to 5) on scGEN's clustering performance. Each bar represents the accuracy (ACC) value under different parameter combinations.

Moreover, our analysis revealed that scGEN has remarkable stability across varied parameter combinations. There was no clear decline in performance when deviating from the optimal settings, especially in the Bell and PBMC 3K datasets, where performance remains consistent despite considerable parameter variations. This level of stability was consistently observed in all datasets, suggesting that scGEN's performance is robust to parameter changes and generalizes well across different biological systems. These results suggest that scGEN is inherently robust and requires minimal parameter tuning, making it a suitable approach for single‐cell RNA sequencing analysis in diverse experimental contexts. In the main experiments, the weighting proportion was fixed at 10%. We additionally evaluated proportions of 5%, 10%, 15%, 20%, 25%, and 30% across eight datasets using ACC, NMI, and ARI. Based on the overall stability of the results and its highest or tied‐highest performance in 11 of the 24 dataset–metric comparisons, 10% was retained as a robust default (Table S8).

### 2.3 Impact of Dual Loss Functions on scGEN Clustering Performance

In our proposed scGEN, we used two independent loss functions to learn the latent representation: the regularized ZINB loss and the structure‐guided hard‐sample learning loss. To systematically assess the contribution of each loss function to overall clustering performance, we carried out a comprehensive ablation study comparing three variants: the complete scGEN model, scGEN without ZINB loss (scGEN w/o ZINB), and scGEN omitting structure‐guided hard‐sample learning loss (scGEN w/o HSL). We evaluated these variations across eight scRNA‐seq datasets, with each experiment repeated twice to ensure reproducibility. Table S2 details the distributions of three performance metrics, ACC, NMI, and ARI, across all datasets.

Each loss component contributes uniquely: ZINB loss models sparsity and count‐based noise typical of single‐cell data, while structure‐guided loss improves sensitivity to rare or ambiguous cell states. The results indicate that our complete scGEN model consistently outperforms both ablated variations. Specifically, the removal of ZINB loss led to substantial performance degradation, with average decreases of 8.37%, 4.55%, and 8.03% in ACC, NMI, and ARI, respectively. Similarly, the absence of structure‐guided hard‐sample learning loss led to performance reductions of 7.64%, 5.35%, and 9.13% in ACC, NMI, and ARI, respectively. The combination of both loss functions raised ACC scores and improved NMI and ARI measures in all datasets. The results confirm that the integration of regularized ZINB loss and structure‐guided hard‐sample learning loss significantly boosts scGEN's clustering performance, with consistent improvements across multiple evaluation metrics and datasets. Because removal of the ZINB loss eliminates the complete ZINB component, this ablation supports the contribution of the overall component but does not isolate the specific effect of stochastic HVG weighting.

### 2.4 scGEN Demonstrates Robust Performance in Complex Cellular Differentiation Trajectories

During cellular development, gene expression patterns often display complex non‐linear dynamics. Some genes display low expression in early stages, peak during intermediate stages, and are subsequently downregulated in terminal stages. To evaluate scGEN's performance in handling sophisticated differentiation trajectories, we employed the Splatter package in R to generate synthetic datasets that specifically represent the intricacies of cellular differentiation trajectories. Splatter is a comprehensive modeling framework that simulates sequential transitions across cellular states, while incorporating key features of scRNA‐seq data, such as technical noise, biological variation, and dropout rates. In our simulation, we generated fifteen distinct datasets, each representing a different level of granularity in the differentiation process. Specifically, we simulated 5000 cells, each containing 5000 genes, with differentiation stages ranging from 2 to 16 (Figure 4A).

**FIGURE 4:**
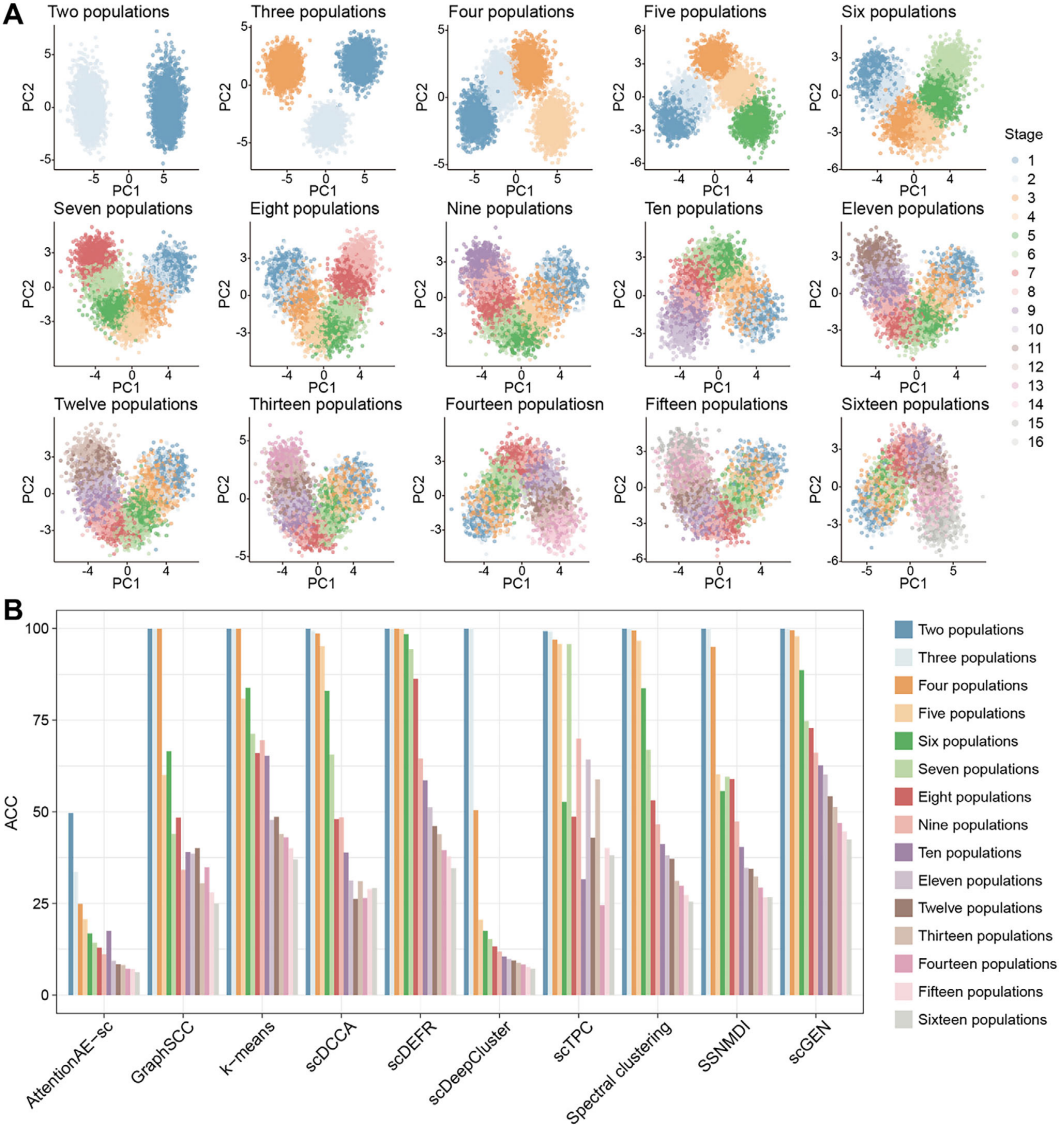
Evaluation of model performance on simulated differentiation trajectories with varying granularities. (A) PCA visualization of fifteen synthetic datasets generated using the Splatter package, representing different stages of cellular differentiation. Each dataset contains 5000 cells with 5000 genes. The number of differentiation stages increases from 2 to 16. Colors indicate different differentiation stages. (B) Comparative performance analysis of different clustering methods across the fifteen synthetic datasets. The bar plot shows the ACC scores for 10 different methods. Each bar represents performance on a specific Splatter dataset, demonstrating how clustering accuracy changes with increasing differentiation complexity. The color‐coding corresponds to the different Splatter datasets as indicated in the legend.

Figure 4B illustrates a comparative analysis of scGEN and nine other clustering methods as the granularity of differentiation stages increases. While some approaches (GraphSCC, scDEFR, scDCCA, and Spectral Clustering) performed better with coarse discretization, they suffered a considerable decline in accuracy with finer‐grained discretization. For example, GraphSCC and scDEFR dropped from near‐perfect performance to below 40% accuracy as the number of stages increased. In contrast, scGEN showed more resilience to performance loss from discretization. Although a slight decrease in performance was observed with increased granularity, the decline was more gradual, and scGEN maintained higher accuracy even under fine‐grained conditions with up to sixteen stages. This consistent high efficiency across various discretization levels is particularly useful when analyzing heterogeneous cell populations, where cell states exist along continuous trajectories rather than as discrete states. This stability stems from scGEN's effective identification and utilization of representative hard samples in single‐cell contexts, highlighting its strength in managing complex cellular differentiation processes.

### 2.5 scGEN Reveals Distinct Developmental Phases and Refined Cell‐Type Assignments in Human Fetal Pituitary Development

To assess the efficacy of our scGEN approach in elucidating complex tissue development, we reanalyzed a published single‐cell RNA‐seq dataset from human fetal pituitaries spanning 7–25 weeks post‐fertilization. Eight methods, excluding AttentionAE‐sc with the lowest accuracy and SSNMDI, were included in this analysis. SSNMDI was excluded as it employs a semi‐supervised framework that utilizes partially labeled cells within the dataset to guide the clustering process. This approach produces predictions only for the unlabeled test subset rather than the complete dataset, making it inappropriate for comparative analysis. All seven methods, except for scDCCA, successfully identified all of the 14 cell types (Figures 5A,B and S1A,B. Notably, four methods, GraphSCC, scDEFR, scDeepCluster, and k‐means, produced inconsistent cell‐type assignments for the mesenchymal cells (MC) population compared to the original annotations (Figures 5C and S1C.

**FIGURE 5:**
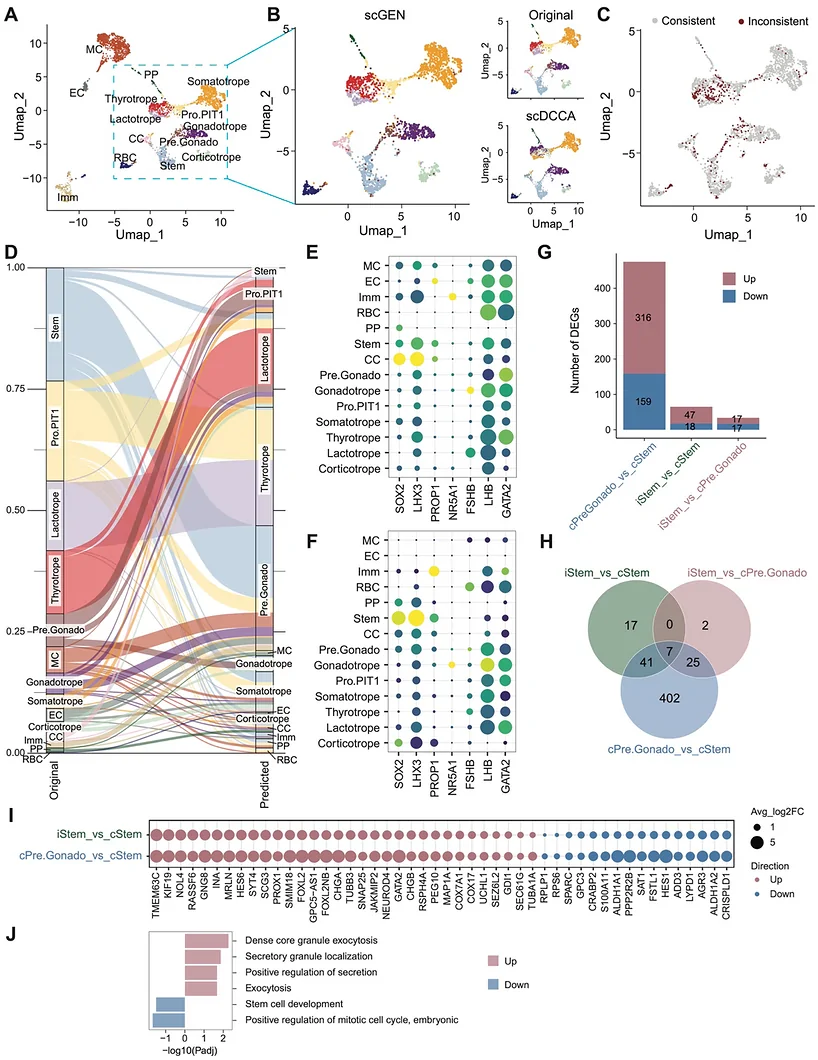
scGEN provides insights into transcriptional dynamics for complex developmental trajectories. (A) UMAP plot, color‐coded for cell type. MC, mesenchymal cells, EC, endothelial cells, Imm, immune cells, PP, pituicytes, CC, cycling cells, RBC, red blood cells. (B) Zoomed‐in UMAP to show the hard samples from scGEN, original, and scDCCA approaches. (C) UMAP plot shows the inconsistent cells identified between the scGEN and original approaches in the clusters. (D) Sankey plots showing the cell types for the inconsistent cells in the original paper and scGEN methods. (E,F) Dot plot showing the canonical marker expression for the cell types defined based on the original and scGEN approach for the inconsistent cells. (G) Bar plot showing the number of differentially expressed genes (DEG) in three comparisons: consistent Pre.Gonado versus consistent stem cells (cPre.Gonado vs. cStem), inconsistent stem cells versus consistent stem cells (iStem vs. cStem), and inconsistent stem cells versus consistent Pre.Gonado (iStem vs. cPre.Gonado). Colors indicate the up‐regulated genes (red) or down‐regulated genes (blue) in each comparison. (H) Venn diagram showing the shared and unique DEGs among three comparisons. (I) Dot plot showing the direction of the shared 48 DEGs between cPre.Gonado vs. cStem and iStem vs. cStem. (J) Functional enrichment analysis of the shared 48 DEGs was performed using Gene Ontology (GO), where colors represent up‐regulated genes (red) or down‐regulated genes (blue) as input.

Here, ‘consistent’ refers to cells whose predicted labels match the original annotations, while ‘inconsistent’ refers to those with revised assignments based on predicted clustering. When we compared the cell‐type scores for consistent and inconsistent MC‐labeled cells across these methods, both groups exhibited the highest MC scores, suggesting that these cells are likely true members of the MC population (Figure S1D). Additionally, although Spectral Clustering and scTPC correctly identified MC cells, Spectral Clustering misclassified Pro.PIT1 and Pre.Gonado cells, while scTPC misclassified gonadotrope and corticotrope cells, highlighting the difficulty of accurately resolving adjacent endocrine lineages (Figure S1A–C).

Interestingly, our scGEN uncovered discrepancies compared to the original annotations (Figure 5C,D); specifically, 369 cells did not conform to their initial labels. Many of these cells, originally classified as stem cells, displayed higher expression of gonadotrope precursor markers (*GATA2*, *NR5A1*, *FSHB*, and *LHB*) and lower levels of typical stem cell markers, suggesting a putative Pre.Gonado‐like transcriptional state (Figure 5E,F). We also observed that some cells previously identified as lactotropes were more accurately defined as thyrotropes. These refinements illustrate scGEN's capacity to resolve ambiguous transcriptional profiles and improve the biological accuracy of cell‐type assignments.

To further characterize these refined annotations, we performed differential expression analyses across three comparisons: consistent Pre.Gonado versus consistent stem cells (cPre.Gonado vs. cStem), inconsistent stem cells versus consistent stem cells (iStem vs. cStem), and inconsistent stem cells versus consistent Pre.Gonado cells (iStem vs. cPre.Gonado). Figure 5G shows that the cPre.Gonado vs. cStem comparison has the most pronounced differences, with 316 upregulated and 159 downregulated genes, while a Venn diagram reveals 48 differentially expressed genes (DEGs) that consistently change in the same direction in both cPre.Gonado vs. cStem and iStem vs. cStem comparisons (Figure 5H,I). Among these shared 48 DEGs, canonical gonadotrope markers such as *GATA2*, *FOXL2*, *CHGA*, *CHGB*, and *NEUROD4* were significantly upregulated, whereas genes typically linked to stemness, including *ALDH1A1*, *ALDH1A2*, *HES1*, and *S100A11*, were downregulated, supporting our reclassification (Figure 5I).

In addition, GO‐based enrichment analysis of these 48 shared DEGs revealed a significant upregulation of secretory functions, such as “dense core granule exocytosis,” “secretory granule localization,” “positive regulation of secretion,” and “exocytosis.” These findings align well with the endocrine features expected of gonadotrope precursors (Figures 5J and S2A,B). Meanwhile, the downregulation of terms like “stem cell development” and “positive regulation of the mitotic cell cycle, embryonic” reflects the loss of proliferative, undifferentiated characteristics as cells commit to a more specialized state (Figure 5J). To avoid relying solely on scGEN‐derived labels, we further evaluated predefined marker signatures associated with Pre.Gonado‐lineage states. The scGEN‐refined ambiguous cells showed stronger Pre.Gonado‐lineage marker activity (Figure S3), supporting their Pre.Gonado‐like transcriptional profile independently of differential genes selected using scGEN labels (Figure 5J). Together, these results underscore the robustness of our scGEN approach in recapitulating known developmental trajectories and its capacity to enhance resolution by refining cell‐type identities.

### 2.6 scGEN Identifies Putative Early Pre.Gonado‐Like States and Associated Developmental Communication Networks

Building on these refined annotations, we next applied scGEN to examine human pituitary development across weeks 7–25, focusing on the identification of ambiguous transitional cells that conventional methods might overlook. scGEN's enhanced sensitivity to boundary cell detection revealed Pre.Gonado cells as early as 7 weeks post‐fertilization, representing a significant advancement over the previously established timeline (Figure 6A). While the original study reported the emergence of Pre.Gonado cells at 8 weeks [35], our scGEN refinements identified a subset of cells originally annotated as stem cells that displayed early gonadotrope precursor characteristics. These findings suggest the possible presence of an earlier Pre.Gonado‐like transcriptional state of reproductive endocrine programming than previously recognized. Cell–cell communication analysis using CellChat [36] provided functional context for the scGEN‐refined annotations by revealing profound differences between original and refined annotations, with Pre.Gonado cells exhibiting the most dramatic changes in sender‐receiver dynamics (Figure 6B). This finding suggests that scGEN's refinements reflect clear biological communication networks rather than clustering artifacts.

**FIGURE 6:**
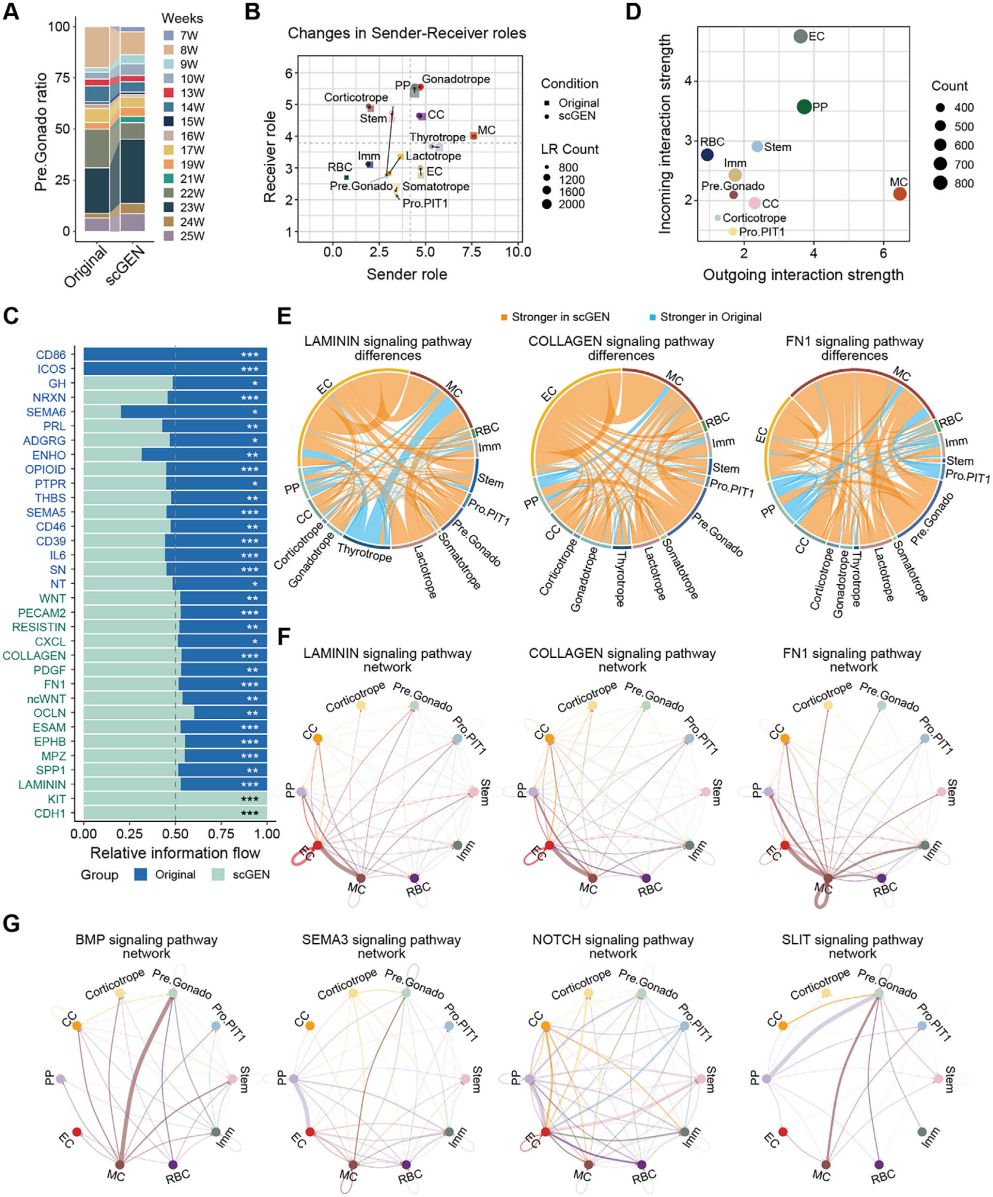
scGEN reveals enhanced cell‐cell communication patterns and earlier Pre.Gonado cell emergence during human pituitary development. (A) Stacked bar chart showing the proportion of Pre.Gonado cells across different weeks (7W‐25W) for original (Zhang et al) and scGEN conditions. (B) Scatter plot depicting changes in sender‐receiver roles between conditions. *X*‐axis shows sender role strength; *Y*‐axis shows receiver role strength. Points represent different cell types, with size indicating LR (Ligand‐Receptor) count and color representing condition. Pre.Gonado cells exhibit the most dramatic changes in sender‐receiver dynamics, validating scGEN's biological accuracy. (C) Heatmap showing relative information flow for various signaling molecules and pathways. Blue indicates stronger expression in the original condition; green indicates stronger expression in scGEN condition. Statistical significance indicated by asterisks (*p < 0.05, **p < 0.01, ***p < 0.001). (D) Bubble plot showing relationship between outgoing and incoming interaction strength at 7‐week timepoints. Bubble size represents count, and different cell types are positioned based on their communication patterns. (E) Chord diagrams comparing signaling pathway differences between conditions for three major ECM pathways: LAMININ, COLLAGEN, and FN1 signaling. Orange links indicate stronger connections in scGEN, blue links indicate stronger connections in the original condition. scGEN reveals extensive reorganization of ECM remodeling pathways with enhanced connectivity patterns. (F) Network diagrams show detailed pathway networks for LAMININ, COLLAGEN, and FN1 signaling pathways at 7 weeks. Nodes represent cell types, edges represent signaling interactions, with edge thickness and color indicating interaction strength. (G) Signaling pathway networks for four critical developmental pathways: BMP, SEMA3, NOTCH, and SLIT at 7 weeks, showing cell‐type‐specific interactions and pathway connectivity patterns. Pre.Gonado cells serve as central nodes in all four networks at 7 weeks, demonstrating coordinated activation of stem‐to‐Pre.Gonado differentiation pathways. MC, mesenchymal cells, EC, endothelial cells, Imm, immune cells, PP, pituicytes, CC, cycling cells, RBC, red blood cells.

Comparative pathway analysis identified 33 signaling pathways with significant differences between original and scGEN annotations, with almost half showing enhanced activity in refined classifications. These findings demonstrate scGEN's ability to unmask biologically relevant signaling networks (Figure 6C). Network analysis across all cells and developmental time points revealed extensive reorganization of ECM remodeling pathways in scGEN‐refined annotations, with LAMININ, COLLAGEN, and FN1 signaling networks showing enhanced connectivity patterns (Figure 6E). Additionally, growth factors and developmental signaling pathways, including PDGF and WNT networks, demonstrated similar enhancement patterns with Pre.Gonado cells actively involved in these communications (Figure S4A). Extracellular matrix pathways showed pronounced enhancements in scGEN‐refined classifications, aligning with ECM's established role in orchestrating organ morphogenesis during development. When focusing specifically on the 7‐week time point, Pre.Gonado cells exhibited distinct communication signatures with both outgoing and incoming interactions among all endocrine lineages, highlighting their critical role during early development (Figure 6D). Network analysis demonstrated complex, multi‐directional communication patterns between Pre.Gonado, Stem, and mesenchymal cell populations (Figure 6F). Among these, PDGF networks showed particularly clear connections originating from various cell types, while WNT and SLITRK networks displayed distributed communication patterns involving multiple cell populations (Figure S4B).

Analysis of classical developmental pathways critical for stem‐to‐Pre.Gonado differentiation revealed Pre.Gonado cells as central nodes in BMP, SEMA3, NOTCH, and SLIT signaling networks at 7 weeks (Figure 6G). Detailed ligand‐receptor analysis confirmed these communication patterns, with Pre.Gonado cells expressing multiple BMP pathway components, including BMP7‐BMPR1A, BMP7‐ACVR1, and BMP2‐BMPR1A receptor pairs (Figure S4C). NOTCH signaling was mediated through DLL3‐NOTCH and JAG1‐NOTCH4 interactions, while SLIT signaling involved SLITRK2‐PTPRS and SLIT2‐ROBO2 receptor pairs (Figure S4D). These molecular interaction patterns provide mechanistic insights into the signaling networks that coordinate early gonadotrope lineage specification. The coordinated activation of these four critical developmental pathways specifically in 7‐week Pre.Gonado cells demonstrates that scGEN's enhanced detection of transitional cell states enables the discovery of previously unrecognized developmental communication networks, showcasing the method's potential for advancing our understanding of complex biological processes through improved computational cell‐type identification.

### 2.7 Independent Histological Validation of a scGEN‐Resolved Intermediate State

To determine whether the developmental ordering implied by scGEN‐refined annotations reflects bona fide in situ developmental states, we performed independent histological validation in human embryos at 6–8 post‐fertilization weeks. We further examined BMPR1A expression and quantified the predicted double‐positive bridging states expected under a progenitor‐to‐gonadotrope progression. Given the limited availability of early‐stage human embryonic pituitary material, these experiments provide valuable tissue‐level support for the scGEN‐refined annotation and its transitional structure.

We analyzed the presumptive pituitary/Rathke's pouch region by H&E and immunofluorescence using lineage markers spanning the progenitor‐to‐gonadotrope axis [35], SOX2 (progenitor/stem), GATA2 (Pre.Gonado), and NR5A1/SF1 (gonadotrope‐lineage), with DAPI counterstaining (Figure 7). Consistent with the developmental framework and the state ordering supported by scGEN, we quantified SOX2^+^ progenitor/stem cells, GATA2^+^ Pre.Gonado cells, and NR5A1^+^ gonadotrope‐lineage cells across developmental stages (Figure 7A,B). The SOX2^+^ compartment decreased; GATA2^+^ Pre.Gonado cells showed a transient profile, and NR5A1^+^ cells increased toward later stages (Figure 7C–G), recapitulating the progenitor‐to‐gonadotrope progression inferred from scGEN‐refined annotations. To move beyond reciprocal abundance changes and directly assess transitional structure, we quantified the predicted bridging populations and observed an early SOX2^+^GATA2^+^ population that declined with development and a later GATA2^+^NR5A1^+^ population that increased toward later stages (Figure S5), supporting temporally ordered transitions through Pre.Gonado. In addition, BMPR1A immunoreactivity was detected within the same Rathke's pouch/pituitary domain enriched for GATA2^+^ cells, with spatial overlap between BMPR1A and GATA2 signals (Figure 7H), providing a receptor‐level context concordant with the BMP‐centered communication programs highlighted by scGEN‐based analyses.

**FIGURE 7:**
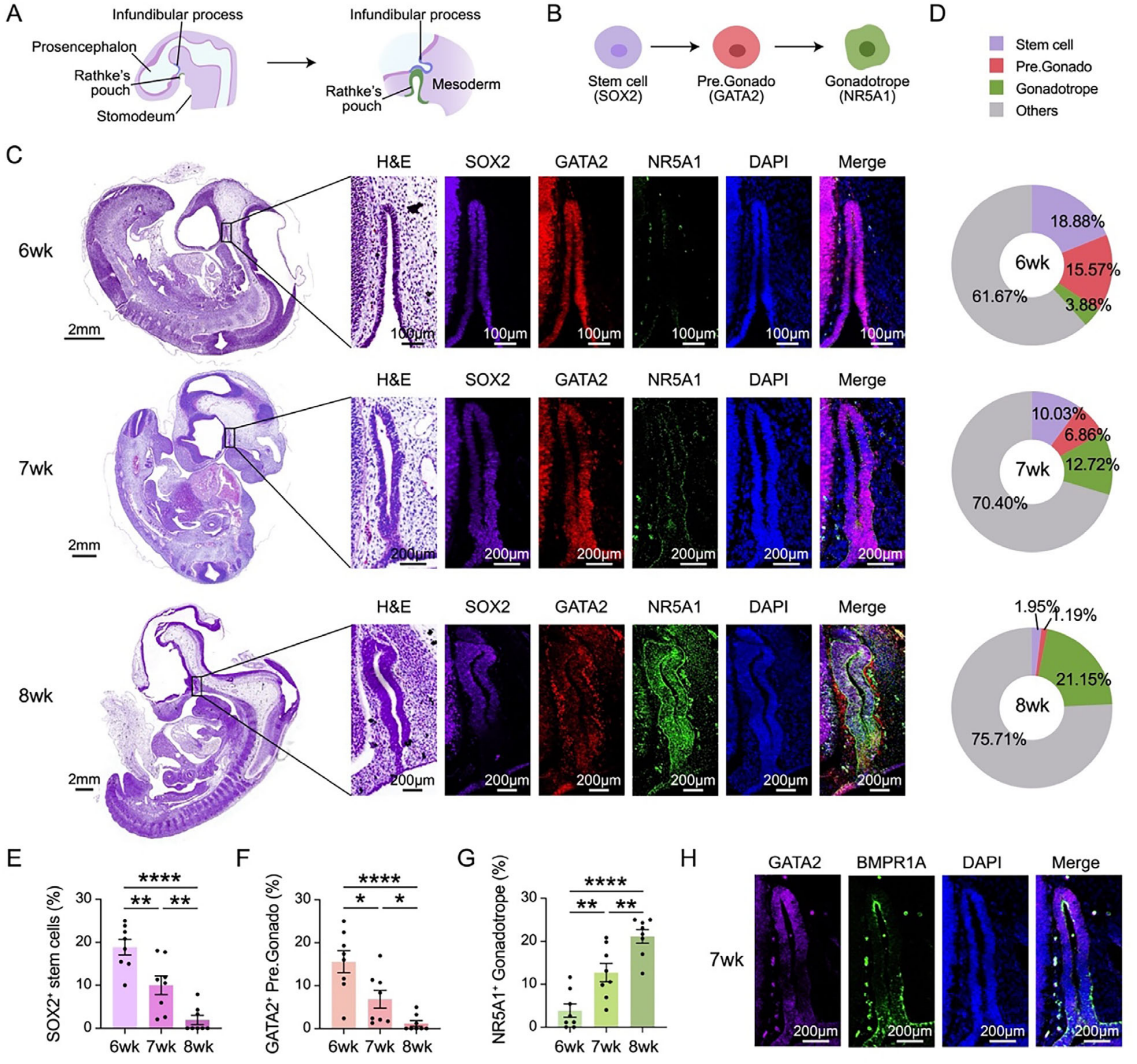
Independent histological validation of a scGEN‐resolved intermediate state during early human pituitary development. (A). Schematic of early pituitary anatomy highlighting Rathke's pouch and the infundibular process. (B). Conceptual framework of the analysed progression from SOX2^+^ progenitor/stem cells through GATA2^+^ Pre.Gonado to NR5A1^+^ gonadotrope‐lineage cells. (C). Representative H&E and immunofluorescence images from early human embryos in the presumptive pituitary/Rathke's pouch region stained for SOX2, GATA2, NR5A1 (SF1), and DAPI. Scale bars as indicated. (D). Donut plots showing the fraction of SOX2^+^ stem/progenitor cells, GATA2^+^ Pre.Gonado cells, NR5A1^+^ gonadotrope‐lineage cells, and other cells each week. (E–G). Quantification of SOX2^+^ stem/progenitor (E), GATA2^+^ Pre.Gonado (F), and NR5A1^+^ gonadotrope‐lineage (G) cells across weeks. Percentages were calculated relative to total DAPI‐detected cells per ROI; data represent individual values and mean ± SD from *n* = 8 ROIs per week. One‐way ANOVA followed by Tukey's post‐hoc tests determined significance. (H). BMPR1A immunostaining and spatial overlap with GATA2 in the morphologically defined Rathke's pouch/pituitary domain.

## 3 Discussion

Accurate identification of cell states, particularly transitional and boundary populations, remains a fundamental challenge in single‐cell transcriptomic analysis. Here, we introduce scGEN, a gene‐aware embedding framework designed to resolve ambiguous cellular identities by selectively emphasizing hard‐to‐classify samples and biologically informative gene signals. By integrating structure‐guided hard‐sample learning with a regularized ZINB objective, scGEN captures both global transcriptional structure and subtle state‐defining features that are often overlooked by conventional clustering approaches. Across eight publicly available scRNA‐seq datasets, scGEN consistently achieves superior clustering accuracy, stability, and biological coherence, demonstrating its capacity to refine cell‐type assignments and uncover previously obscured intermediate states. Our sensitivity analysis further suggests that α = 1 and γ = 1 provide robust default settings for most routine applications. For datasets with substantial heterogeneity, rare populations, or extensive transitional states, a small grid search around these values may further optimize clustering performance.

scGEN addresses key limitations that cause existing methods to struggle with transitional and boundary cell identification [37, 38]. Our structure‐guided hard‐sample learning targets ambiguous cells with overlapping transcriptional profiles that conventional methods often misclassify, using adaptive weighting based on clustering uncertainty rather than treating all samples equally. The novelty of scGEN does not lie in proposing hard‐sample mining as a general concept, but in redesigning it for the specific characteristics of scRNA‐seq clustering. Unlike computer vision methods that rely on image augmentations and spatial patterns, scGEN is tailored to scRNA‐seq data through three domain‐specific designs: (i) a dual‐encoder jointly modeling gene expression and cell–cell graph topology; (ii) a regularized ZINB loss with random HVG weighting to handle sparsity and noise; and (iii) a structure‐guided hard‐sample loss that uses pseudo‐labels and joint similarity to resolve ambiguous cluster boundaries. These are not direct transfers from computer vision, but targeted solutions for single‐cell clustering challenges. The regularized ZINB framework with selective gene emphasis moves beyond relying solely on HVGs by incorporating expression levels and applying differential weighting to gene subsets, which helps to detect subtle signals that standard approaches may overlook. Additionally, our dual‐encoder architecture with adaptive weighting captures cellular relationships through parallel processing of both expression profiles and structural information, improving upon methods that process these signals sequentially. Random sampling was used to preserve the unsupervised and label‐agnostic nature of scGEN. Function‐based selection may bias the model toward known pathways or annotated cell types, whereas variance‐based selection is already incorporated during HVG preprocessing and dropout‐rate‐based selection may be confounded by technical sparsity. Therefore, random sampling within the HVG space provides an unbiased stochastic perturbation for feature‐level regularization. The stochastic HVG‐weighting procedure was not directly compared with standard dropout, L2 regularization, or feature subsampling. Systematic comparisons with these alternative strategies will be valuable for further evaluating its relative performance. Standard contrastive learning relies on data augmentations to define positive pairs and uniformly contrasts samples to learn general representations. In contrast, scGEN constructs pairs using clustering pseudo‐labels and joint similarities, and applies an adaptive weighting matrix that emphasizes hard samples, cells with high similarity but different pseudo‐labels, or low similarity but the same pseudo‐label. Thus, the loss focuses optimization on refining cluster boundaries rather than indiscriminately contrasting all pairs. These methodological contributions work together to enable better identification of ambiguous cell populations and enhance biological interpretability.

Beyond methodological advances, scGEN reveals important biological insights that demonstrate its practical value for developmental biology research. In the fetal pituitary atlas, scGEN identifies a putative Pre.Gonado‐like population at 7 weeks post‐fertilization, the earliest sampled stage, extending our refined cell‐type assignments in human pituitary development. While our first phase of analysis demonstrated that scGEN successfully reclassified ∼10% of the total cells based on their expression of gonadotrope precursor markers, differential expression and GO enrichment analyses were used to characterize the molecular programs associated with the scGEN‐refined Pre.Gonado‐like cells, rather than as independent validation of the label reassignment, the cell–cell communication analysis further revealed the broader biological significance of these refinements. In addition, BMPR1A, a key receptor mediating BMP signaling during pituitary morphogenesis, overlapped with the GATA2^+^ Rathke's pouch domain, providing receptor‐level validation consistent with the BMP‐centered signaling programs identified by communication analysis.

To test whether the scGEN‐refined annotations reflect bona fide in situ progression, we performed independent histological validation in embryos spanning 6–8 weeks post‐fertilization. Immunostaining in the presumptive pituitary/Rathke's pouch region recapitulated the expected developmental hierarchy defined by SOX2, GATA2, and NR5A1 expression. SOX2, marking multipotent pituitary progenitors, progressively contracted, while a transient GATA2^+^ population, consistent with early gonadotrope‐committed precursors, emerged and subsequently gave rise to expanding NR5A1^+^ gonadotrope‐lineage cells. The stage‐dependent bridging populations, characterized by declining SOX2^+^GATA2^+^ intermediates and increasing GATA2^+^NR5A1^+^ transitional cells, support a temporally ordered progression through the Pre.Gonado state predicted by scGEN. Although the immunofluorescence analysis cannot directly trace the exact 369 cells identified in the scRNA‐seq dataset, it provides orthogonal tissue‐level evidence that GATA2^+^ Pre.Gonado‐lineage cells are present in the developing pituitary/Rathke's pouch region during 6–8 weeks. The histological validation should be interpreted as orthogonal tissue‐level support for the presence of GATA2^+^ Pre.Gonado‐lineage cells during early pituitary development, rather than direct validation of the precise identity of individual cells reassigned by scGEN in the scRNA‐seq dataset. Given the limited availability of early‐stage human embryonic pituitary tissue, these findings provide a rare in situ validation layer that strengthens the biological grounding of the scGEN‐refined annotations. The coordinated activation of multiple signaling networks, including BMP morphogenetic gradients [39], NOTCH‐mediated cell fate decisions [40], and ECM‐mediated positional cues [41, 42], suggests that early gonadotrope specification involves sophisticated molecular coordination that was previously unrecognized in human development.

By identifying these intricate developmental programs, scGEN provides a powerful framework for linking computational refinement directly to previously unresolvable biological mechanisms. Consistent with this refined trajectory, the extensive ligand‐receptor networks involving PDGF [43] and WNT pathways [44, 45] reveal that early reproductive endocrine programming requires complex growth factor coordination that may be vulnerable to environmental disruptions during critical developmental windows. These findings illustrate how improved computational resolution directly enables discovery of previously obscured developmental mechanisms. This temporal refinement, achieved with scGEN, of gonadotrope lineage emergence establishes a previously unrecognized critical period during which genetic or environmental perturbations could disrupt reproductive axis establishment, with potential lifelong consequences for fertility and hormonal regulation. This finding builds upon our demonstrated capability to detect ambiguous transitional cells and illustrates how methodological improvements in clustering can lead to fundamental advances in understanding human development. In doing so, scGEN not only enhances the resolution of cell‐state discovery but also establishes a paradigm for leveraging computational innovation to identify critical developmental susceptibilities with direct relevance to human health and disease.

Our systematic focus on the previously underexplored challenge of ambiguous cell identification shows that targeted hard‐sample learning can improve both clustering accuracy and biological discovery. A limitation of scGEN is its reliance on pseudo‐labels derived from clustering, which may propagate early errors in very noisy settings. Because the apparent earlier detection of Pre.Gonado‐like cells may be influenced by sampling depth, batch effects, and analytical resolution, this result should be interpreted as a computationally supported refinement rather than definitive evidence of a revised developmental onset. Further validation using larger developmental series and spatially resolved transcriptomic data will be needed to confirm the precise timing of Pre.Gonado emergence. In the future, we plan to extend our framework to incorporate spatial and temporal single‐cell data analysis and integrate additional biological prior knowledge, laying a solid foundation for advancing our understanding of cellular heterogeneity and development in complex biological systems.

## 4 Methods

### 4.1 Mathematical Framework and Joint Feature Encoding in scGEN

To establish the mathematical foundation for scGEN, we define the variables used in this work, as summarized in Figure 1 and Table S5. The gene expression matrix $X\in R^{N\times D}$ is derived from scRNA‐seq, where the element *X_ij_* denotes the expression level of the *j‐th* gene in the *i‐th* cell. The relationships between cells are represented as an undirected graph G={V,E}, where V represents nodes (cells), E represents edges (connections between cells), and N is the total number of cells. The degree matrix D is defined as D = $\mathrm{diag}(d_{1},d_{2},\text{…},d_{N})$, an *N*  × *N* diagonal matrix in which each element corresponds to the number of connections for the respective node. The adjacency matrix A encodes the direct links between nodes: *a_ij_* = 1 if there is a direct connection between nodes *v_i_* and *v_j_*,​ otherwise *a_ij_* ​ = 0.

To encode attribute and structural information, the K‐nearest neighbors (KNN) algorithm is used to construct a graph that identifies the top *k* neighbors for each cell. If the cell *x_i_* is adjacent to *x_j_*, and *x_j_* is adjacent to *x_k_*, it follows that *x_i_* and *x_k_* may also share a certain degree of similarity. The Pearson correlation coefficient, given in Equation (1), is used to calculate the linear relationship between cells:

(1) $$P_{ij}=\frac{\sum_{k=1}^{d}\left(X_{ik}-{\bar{X}}_{i}\right)\left(X_{jk}-{\bar{X}}_{j}\right)}{\sqrt{\sum_{k=1}^{d}{\left(X_{ik}-{\bar{X}}_{i}\right)}^{2}\sum_{k=1}^{d}{\left(X_{jk}-{\bar{X}}_{j}\right)}^{2}}}$$

Here, X_*ik*_ represents the *k*‐th cell in the *i*‐th row, and ${\bar{X}}_{i}$​ is the average value for the *i* − th row. The adjacency matrix A is then defined based on the calculated Pearson correlation distances (Equation 2):

(2) $$A={\left(a_{ij}\right)}_{N\times N}=\left\{\begin{array}{cc} 1, & \mathrm{if}\left(v_{i},v_{j}\right)\in E\\ 0, & \mathrm{else} \end{array}\right.$$

Once the graph is constructed, scGEN processes the input data {**X**, **A**} using parallel encoders. Instead of using a single neural network F(·) to compress features, scGEN employs two parallel neural networks that extract features independently to capture different aspects of semantic information. The attribute embeddings are computed as:

(3) $$H_{A1}=F_{a}^{1}\;\left(X\right);\;\;H_{A2}=F_{a}^{2}\;\left(X\right)$$

where $F_{a}^{1}$ and $F_{a}^{2}$ are the two parallel attribute encoders, and **H**_*A*1_and **H**_*A*2_ are their corresponding attribute embeddings. Similarly, structural information is encoded as:

(4) $$H_{S1}=F_{s}^{1}\;\left(A\right);\;\;H_{S2}=F_{s}^{2}\;\left(A\right)$$

where $F_{s}^{1}$​ and $F_{s}^{2}$ are structural encoders and **H**_*S*1_​ and **H**_*S*2_​ are structural embeddings.

To integrate these features, scGEN employs two strategies: (1) constructing a joint similarity measure $S_{J}\left(\cdot \right)$, that combines attribute similarity $S_{ATT}\left(\cdot \right)$ and the structural similarity $S_{STR}\left(\cdot \right)$ derived from the representation reconstruction H^T^H, defined as:

(5) $$S_{J}=\beta S_{ATT}+\left(1-\beta \right)S_{STR}$$

where *β* is a learnable parameter balancing the contributions of attribute and structural information; and (2) generating joint representations $H_{J}$ that integrate attribute and structural embeddings:

(6) $$H_{J}=\frac{1}{4}\;\left(H_{A1}+H_{A2}+H_{S1}+H_{S2}\right)$$

These strategies ensure that scGEN effectively captures both attribute and structural information, enhancing its ability to identify meaningful relationships between cells.

### 4.2 Structure‐Guided Hard‐Sample Learning in scGEN for Enhanced Single‐Cell Clustering

This section explains how the joint similarity metric is utilized to guide the network's focus on hard samples. First, k‐means clustering is performed on the encoded joint representation $H_{J}$ ​ to generate initial clustering results, which serve as pseudo‐labels (P) for each cell. This process is formalized as:

(7) $$f_{k}:H_{J}\to R^{k}$$

where *f*_k_ maps the joint representation to k clusters. Inspired by prior work, the distance of each sample to the cluster centers is computed, and the top *N*′ samples, deemed reliable, are designated as the reliable set **R**. These reliable samples are subsequently used to compute sample pair weights. The number of reliable samples was determined using a fixed high‐confidence rate τ. Specifically, for a dataset containing N cells, we selected (1− τ) × 100% reliable samples with the smallest normalized distances to their assigned cluster centers. We used τ = 0.9 throughout this study, and this value was kept fixed across all datasets rather than optimized in a dataset‐dependent manner. The reliable set was updated iteratively during training and used to guide the adaptive sample‐pair weighting. Based on the pseudo‐labels P, the pseudo‐labels for sample pairs (**Q**) are calculated as:

(8) $$Q_{ij}=\left\{\begin{array}{cc} 1, & \mathrm{if}\;P_{i}=P_{j}\\ 0, & \mathrm{if}\;P_{i}\;\neq \;P_{j} \end{array}\right.$$

where **Q**_*ij*_ indicates the relationship between samples *i* and *j*: a value of 1 indicates a positive pair, while 0 denotes a negative pair. Using the constructed **Q** and **R**, the sample pair weight measurement M is defined as:

(9) $$M\;\left(i,j\right)=\left\{\begin{array}{cc} 1, & \mathrm{if}\;i,j\notin R\\ {\left|Q_{ij}-\mathrm{Norm}\left(S_{J}\left(i,j\right)\right)\right|}^{\gamma }, & \mathrm{otherwise} \end{array}\right.\;$$

Through this weighted measurement M, the network is guided to prioritize learning from challenging samples. Specifically, when neither sample *i* nor *j* belongs to the reliable set, the original weights remain unchanged ($M_{ij}=1$). As for reliable samples, we dynamically adjust this weight using the discrepancy between pseudo‐labels and attribute‐structural similarity $S_{J}$. In scGEN, hard samples are defined as those exhibiting either (i) low transcriptional similarity to other cells within their own predicted cluster, or (ii) high similarity to cells belonging to different clusters—both cases indicating ambiguous or transitional cellular identities. In both cases, as the similarity becomes more contradictory to the expected relationship (defined by **Q**_*ij*_), the weight M increases proportionally. As a result, hard samples are assigned higher weights, directing the network to focus on them more effectively.

Using the computed sample pair weights M and the attribute‐structural similarity $S_{J}$​, we propose a structure‐guided hard‐sample contrastive loss:

(10) $$L_{H}=\frac{1}{N}\;\sum_{i,j=1}^{N}-log\left(\frac{e^{M\left(i,i\right)\cdot S_{J}\left(i,i\right)}}{e^{M\left(i,i\right)\cdot S_{J}\left(i,i\right)}+\sum_{i\neq j}e^{M\left(i,j\right)\cdot S_{J}\left(i,j\right)}}\right)$$

Compared to traditional contrastive loss, our designed structure‐guided hard‐sample contrastive loss effectively leverages attribute and structural information from the cellular matrix. Additionally, by incorporating adaptive weighting, this approach ensures the network focuses more on hard sample pairs, thereby enhancing the representation and learning of challenging cellular data.

### 4.3 Regularized ZINB Loss in scGEN for Robust Single‐Cell Clustering

The ZINB distribution has gained significant traction in single‐cell clustering due to its ability to model the sparse and over‐dispersed nature of single‐cell data effectively. The standard ZINB framework employs three multilayer perceptrons to denoise the data, with a distinct fully connected layer to estimate the mean (M), dispersion (Θ), and dropout probability (Π):

$$\Pi =\;\mathrm{sigmoid}\left(W_{\pi }F_{d}\left(H_{J}\right)\right)$$

(11) $$M\;=\exp\left(W_{\mu }F_{d}\left(H_{J}\right)\right)$$

$$\Theta =\exp\left(W_{\theta }F_{d}\left(H_{J}\right)\right)$$

However, these conventional implementations of ZINB models, while effective for single‐cell data, often struggle with the high feature‐to‐sample ratio characteristic of scRNA‐seq data, leading to potential overfitting. To address this, we introduce a weighted ZINB framework that stochastically adjusts the contributions of selected HVGs during loss calculation. Our method modifies the standard ZINB framework by applying differential weighting to gene subsets during loss calculation. The gene expression adjustment is defined as:

(12) $${\tilde{X}}_{ij}=\left\{\begin{array}{cc} X_{ij}, & \;\mathrm{if}\;j\notin \sigma_{1}\;*d\\ \sigma_{2}*X_{ij} & \mathrm{else} \end{array}\right.$$

where σ_1_ represents the proportion of genes receiving emphasis, and d is the total number of genes. The weight index σ_2_ further amplifies the contribution of selected HVGs in the model. The selection of genes for emphasis can follow multiple strategies, with our baseline implementation using a Monte Carlo approach that emphasizes a random 10% of features. In our implementation, feature weighting is applied after preprocessing and highly variable gene selection. Specifically, the model receives the 2000 selected HVGs as input, and the adaptive ZINB weighting is performed only within this selected input feature space rather than across all genes in the raw expression matrix. The weighting proportion was fixed at 10% in all main experiments. In the baseline implementation, 10% of the selected input HVGs are emphasized during the ZINB loss calculation, whereas the remaining HVGs retain the baseline weight. This strategy provides feature‐level regularization and reduces the model's tendency to overfit dominant expression programs.

The weighted ZINB distribution is then computed using the weighted expression $\tilde{X}$:

(13) $$\mathrm{NB}\;\left(\tilde{X}|\mu ,\theta \right)=\frac{\Gamma \left(\tilde{X}+\theta \right)}{\tilde{X}!\Gamma \left(\theta \right)}\;{\left(\frac{\theta }{\theta +\mu }\right)}^{\theta }{\left(\frac{\mu }{\theta +\mu }\right)}^{\tilde{X}}$$

(14) $$ZINB\;\left(\tilde{X}|\pi ,\mu ,\theta \right)=\pi \delta_{0}\left(\tilde{X}\right)+\left(1-\pi \right)\mathrm{NB}\left(\tilde{X}\right)$$

where *π*, *μ*, and *θ* represent the dropout rate, mean, and dispersion, respectively. Using this regularized ZINB distribution, the negative log‐likelihood is employed to calculate the final objective function:

(15) $$L_{Z}=-\log\left(\mathrm{ZINB}\left(\tilde{X}|\pi ,\mu ,\theta \right)\right)$$

This selective emphasis stochastically alters the reconstruction contributions of selected HVGs during training. We therefore describe it as a stochastic feature‐level weighting strategy rather than a demonstrated anti‐overfitting mechanism. The present study does not directly compare this procedure with standard dropout, L2 regularization, or feature subsampling. By systematically emphasizing randomly selected gene subsets, the approach reduces the model's tendency to specialize in dominant expression patterns, instead promoting balanced learning across the feature space.

Finally, the regularized ZINB loss is combined with the structure‐guided hard‐sample contrastive loss ($L_{H}$​) to jointly optimize the cell embedding:

(16) $$L=L_{H}+\alpha L_{Z}$$

where α is a trade‐off hyperparameter that balances the contributions of the two modules. The combined objective jointly optimizes ZINB reconstruction and structure‐guided hard‐sample discrimination to improve the learned cell representations.

### 4.4 Benchmark Datasets and Experimental Setup

We collected eight scRNA‐seq datasets from publicly available platforms (Table S6), which included both human and mouse tissue types from multiple platforms, each with well‐defined “gold‐standard” cluster labels. Two datasets were obtained from Hrvatin et al. [46]., which explored transcriptional responses in the mouse visual cortex using high‐throughput droplet‐based scRNA‐seq. The PBMC 3K dataset, often used as a benchmarking resource, was obtained from 10x Genomics. The Zhang dataset, consisting of 4113 cells from human embryonic pituitaries, was collected by Zhang et al. [35]. to understand pituitary development. Additionally, four datasets (originally from Bell et al. [47]., Schwalbe et al. [48]., Scala et al. [49]., and Savas et al. [50]., respectively) were downloaded from the Single Cell Portal (SCP). The Bell dataset focuses on amyotrophic lateral sclerosis (ALS) neurons generated from the Phox2a lineage, with single‐nucleus RNA sequencing to explore neuronal diversity in ALS [47]. The Schwalbe dataset mainly targets ventrolateral medulla (VLM) neurons, particularly adrenergic/noradrenergic and spinally‐projecting subtypes [48]. The Scala dataset examines transcriptional diversity in the brain [49], while the Savas dataset highlights T‐cell heterogeneity in human breast cancer [50]. The number of cells in these datasets ranged from 206 to 5759 cells, with detailed characteristics summarized in Table S6.

We then compared scGEN with nine other approaches, including six deep learning‐based methods (AttentionAE [27], GraphSCC [28], scDEFR [29], scDeepCluster [30], scDCCA [12], and scTPC [31]) and three classical clustering methods (k‐means, Spectral Clustering, and SSNMDI [32]) (Table S7). The scGEN model was implemented in Python (version 3.10.8) with the PyTorch framework (version 2.5.1). The model was trained for 400 epochs with 20 independent runs to ensure stability and reproducibility. Two hyperparameters, α and γ, were optimized by grid search, including α values spanning {0.01, 0.1, 1, 10, 100} and γ values ranging from {1, 2, 3, 4, 5}. Based on the parameter sensitivity analysis, we recommend α = 1 and γ = 1 as default starting values for applying scGEN to new datasets. For complex or unfamiliar datasets, users may optionally perform a small local grid search around α ∈ {0.1, 1, 10} and γ ∈ {1, 2, 3, 4, 5}. All analyses were performed on a Rocky 9 workstation with an Intel Xeon Gold 6238R CPU and two NVIDIA Tesla V100S graphics cards.

### 4.5 Comparison with Classical Single‐Cell Clustering Methods

To evaluate scGEN's performance against widely used classical clustering approaches, we conducted comprehensive comparisons with Seurat (version 5.3.0) and Scanpy (version 1.11.4) pipelines. For Seurat, we used the standard workflow with graph‐based clustering using the Louvain algorithm. For Scanpy, we employed the standard preprocessing pipeline with Leiden clustering. To ensure fair comparisons, we tested multiple resolution parameters for each method across all eight datasets: resolution parameters for both Seurat and Scanpy ranged from 0.1 to 2.0, depending on dataset complexity. For each dataset, we selected five resolution parameters that produced the number of clusters closest to the ground truth annotations, and reported the best performance achieved across all tested resolutions. Because this resolution selection is label‐informed, these results should be interpreted as an optimistic upper bound for the classical pipelines in this benchmarking setting. All analyses were performed using default parameters unless otherwise specified.

### 4.6 Validation of Temporal and Functional Dynamics Using scGEN

To evaluate clustering performance, we used three widely recognized metrics: clustering ACC, NMI [34], and ARI [33]. ACC quantifies how well predicted clusters align with ground truth classes by finding the optimal one‐to‐one mapping between predicted and true labels, with values ranging from 0 to 1, where higher scores indicate better alignment. NMI measures the mutual dependence between predicted and true labels, normalized to account for differences in cluster sizes, and produces values between 0 and 1. ARI assesses the similarity between predicted and true labels, adjusting for chance agreement, with scores ranging from −1 to 1; values close to 1 reflect strong concordance, 0 indicates random labeling, and negative values suggest less agreement than expected by chance. Together, these metrics provide a comprehensive evaluation framework for assessing clustering performance.

### 4.7 Exploration of Temporal and Functional Dynamics in Human Fetal Pituitary Development Using scGEN Modes

The single‐cell RNA‐seq dataset from Zhang et al. [35], which consisted of 4113 cells from 21 fetal pituitaries (7–25 weeks), was further used to explore complex tissue development. We used eight different algorithms (GraphSCC [28], scDEFR [29], scDeepCluster [30], scDCCA [12], k‐means, Spectral Clustering, scTPC [31], and scGEN) for cell‐type classification. Differential expression analysis was performed with FindMarker in Seurat [51], and Gene Ontology (GO) enrichment analysis was conducted using our in‐house richR package (version 0.0.31) (https://github.com/hurlab/richR) with GO annotations.

### 4.8 Cell–Cell Communication Network Analysis

Cell–cell communication analysis was performed using CellChat [36] to compare communication networks between original cell annotations and scGEN‐refined annotations. CellChat objects were created for both annotation sets using the human ligand‐receptor database (CellChatDB.human). Communication probabilities were computed using default parameters.

Pathway‐specific information flow was calculated as the sum of communication probabilities for each pathway normalized by total communication strength. Differential network analysis was performed to identify pathways with enhanced or reduced activity between original and refined annotations. For focused analysis of 7‐week Pre.Gonado cells, we subset the data to include only 7‐week timepoints and analyzed ECM remodeling (COLLAGEN, LAMININ, FN1), growth factor signaling (PDGF, WNT), and developmental pathways (BMP, SEMA3, NOTCH, SLIT). Statistical significance was determined using CellChat's built‐in permutation tests, with *p* < 0.05 considered significant. Cell–cell communication figures were plotted using our in‐house CellDiff package (version 0.1.3) (https://github.com/guokai8/CellDiff).

### 4.9 Tissue Collection and Ethical Statement

Human embryonic tissue samples (6–8 weeks post‐fertilization) were obtained from Guangzhou Women and Children's Medical Center. This study was conducted in accordance with the Declaration of Helsinki and approved by the Institutional Review Board (IRB) of the Medical Ethics Committee of Guangzhou Women and Children's Medical Center (Approval No. [2024]306A01). Written informed consent was obtained from all participants prior to sample collection. The embryonic tissues were derived from patients undergoing voluntary termination of pregnancy. Following the procedure, the products of conception were immediately transferred to the Department of Pathology, where 6–8 weeks of embryonic tissues were identified and fixed in 10% neutral‐buffered formalin (NBF).

### 4.10 Histological Processing and H&E Staining

After fixation in 10% NBF for at least 48 h, tissues were dehydrated through a graded ethanol series, cleared in xylene, and embedded in paraffin wax. Serial sections (10 µm thick) were cut and dried at 65°C for 2 h to ensure adhesion. Hematoxylin and Eosin (H&E) staining was performed using a commercial kit (Beyotime, #C0105S) following the manufacturer's instructions. Briefly, sections were deparaffinized in xylene and rehydrated through a descending ethanol gradient (100% to 75%). The slides were stained with hematoxylin for 5 min, rinsed in running tap water, and counterstained with eosin for 30–60 s. Finally, the sections were dehydrated, cleared, and mounted with permanent mounting medium. Brightfield images were acquired with a Zeiss Axioscan 7 slide scanner (20× objective) and analyzed in ImageJ (NIH).

### 4.11 Multiplex Immunofluorescence (mIF) via TSA

To enable simultaneous detection of multiple markers from the same host species, Tyramide Signal Amplification (TSA)‐based mIF was performed on consecutive paraffin sections. Following deparaffinization and rehydration, heat‐induced epitope retrieval (HIER) was performed in Tris‐EDTA buffer (pH 9.0) at 95°C for 20 min. Non‐specific binding was blocked for 30 min at room temperature (RT). Sections were then incubated with primary antibodies for 1 h at RT. The primary antibodies used (all from Proteintech) included: anti‐SOX2 (#11064‐1‐AP, 1:500), anti‐NR5A1 (#18658‐1‐AP, 1:500), anti‐GATA2 (#11103‐1‐AP, 1:500), and anti‐BMPR1A (#12702‐1‐AP, 1:500).

Signal detection was achieved using Multi‐rAb Polymer HRP‐Goat Anti‐Rabbit secondary antibody (30 min, RT), followed by incubation with TSA reagents (Proteintech, #PK10033) for 10 min at RT. For sequential multi‐target labeling, the previously bound antibody‐HRP complex was stripped via HIER (Tris‐EDTA, pH 9.0) after each staining round. This cycle was repeated for each target marker. Nuclei were counterstained with DAPI, and slides were mounted using an anti‐fade medium. Fluorescence images were acquired with a Zeiss LSM980 confocal microscope (20× or 40× objectives). Slides were analyzed using QuPath open‐source software (version 0.5.1) [52] for the quantification of positively stained cells as previously described [53]. Adjacent sagittal H&E‐stained sections were used as an anatomical guide to identify the early pituitary/Rathke's pouch region on the corresponding immunofluorescence sections. Based on the morphology of the presumptive pituitary/Rathke's pouch domain, 8 regions of interest (ROI) were selected across the entire region per group (week) by an experienced researcher using the annotation function in QuPath. Within each ROI, nuclei were identified and segmented using the DAPI channel, followed by cell phenotyping according to the fluorescence signals of SOX2, GATA2, and NR5A1 markers. This workflow allowed the quantification of SOX2^+^ progenitor/stem cells, GATA2^+^ Pre.Gonado cells, NR5A1^+^ gonadotrope‐lineage cells, and SOX2^+^GATA2^+^ or GATA2^+^NR5A1^+^ double‐positive cells within the presumptive pituitary/Rathke's pouch region.

### 4.12 Statistical Analysis

The data processing pipeline includes data normalization with Seurat's “NormalizeData” function [51] and feature variance filtering by selecting 2000 HVGs using the Python package Scanpy [54]. ACC, NMI, and ARI were calculated in Python (version 3.10.8) and are presented as mean ± standard deviation (SD). Statistical significance of cell–cell communication was assessed using the Wilcoxon test. GraphPad Prism (version 10.4.1) was used to perform mIF image statistical analysis on the exported data. One‐way ANOVA followed by Tukey's post‐hoc tests was applied. Significance levels were defined as *p < 0.05, **p < 0.01, and ***p < 0.001.

## Author Contributions

**Fang Huang**: investigation, resources, data curation, formal analysis, methodology, software, visualization, writing – original draft, writing – review and editing. **Dayu Hu**: investigation, resources, formal analysis, methodology, software. **Yaqi Dai**: resources, validation, visualization. **Aoyi Geng**: formal analysis, data curation, visualization. **Renxiang Guan**: methodology, writing – original draft. **Hao Quan**: methodology, writing – original draft. **Brett A. McGregor**: writing – original draft, writing – review and editing. **Zhihan Wang**: writing – original draft, writing – review and editing, visualization. **Nadeem Khan**: writing – review and editing. **Hongsheng Zhang**: resources, writing – review and editing. **Jinghua Zhang**: writing – review and editing. **Mingyang Xia**: resources, writing – review and editing. **Xiaoqiong Gu**: resources, writing – review and editing. **Di Che**: resources, writing – review and editing. **Junguk Hur**: methodology, data curation, supervision, funding acquisition, writing – review and editing. **Xiaoyu Cui**: methodology, supervision, writing – review and editing. **Kai Guo**: conceptualization, investigation, resources, validation, supervision, funding acquisition, writing – review and editing.

## Conflicts of Interest

The authors declare no conflicts of interest.

## Supporting information




**Supporting File**: advs77746‐sup‐0001‐SuppMat.docx.

## Data Availability

The raw scRNA‐seq data used in this study are available from either Single Cell Portal (IDs: SCP2337, SCP2306, SCP2674, and SCP2331) or Gene Expression Omnibus (IDs: GSE102827, GSE142653). To facilitate broad adoption, we have made scGEN freely available as a Python package at https://github.com/hurlab/scGEN and permanently archived at https://zenodo.org/records/16949673.

## References

[1] A. A. Kolodziejczyk , J. K. Kim , V. Svensson , J. C. Marioni , and S. A. Teichmann , “The Technology and Biology of Single‐Cell RNA Sequencing,” Molecular Cell 58, no. 4 (2015): 610–620, 10.1016/j.molcel.2015.04.005. 26000846

[2] D. Hu , Z. Dong , K. Liang , H. Yu , S. Wang , and X. Liu , “High‐Order Topology for Deep Single‐Cell Multiview Fuzzy Clustering,” in IEEE Transactions on Fuzzy Systems (IEEE, 2024), 4448–4459.

[3] Q. Li , N. An , C. Liu , et al., “Single‐Cell BCR and Transcriptome Analysis Reveals Peripheral Immune Signatures in Patients with Thyroid‐Associated Ophthalmopathy,” Aging 16, no. 9 (2024): 8217. 10.18632/aging.205814 PMC11132005 38728262

[4] H. Keren‐Shaul , A. Spinrad , A. Weiner , et al., “A Unique Microglia Type Associated With Restricting Development of Alzheimer's Disease,” Cell 169, no. 7 (2017): 1276–1290.e17, 10.1016/j.cell.2017.05.018. 28602351

[5] D. Grün , A. Lyubimova , L. Kester , et al., “Single‐Cell Messenger RNA Sequencing Reveals Rare Intestinal Cell Types,” Nature 525, no. 7568 (2015): 251–255. 10.1038/nature14966 26287467

[6] C. C. Brown , H. Gudjonson , Y. Pritykin , et al., “Transcriptional Basis of Mouse and Human Dendritic Cell Heterogeneity,” Cell 179, no. 4 (2019): 846–863.e24, 10.1016/j.cell.2019.09.035. PMC6838684 31668803

[7] D. Hu , K. Liang , S. Zhou , W. Tu , M. Liu , and X. Liu , “VisANT: An Integrative Framework for Networks in Systems Biology,” Briefings in Bioinformatics 9, no. 4 (2023): 317–325, 10.1093/bib/bbn020. PMC2743399 18463131

[8] D. Hu , R. Guan , K. Liang , et al., “MLSNet: A Deep Learning Model for Predicting Transcription Factor Binding Sites,” Briefings in Bioinformatics 25, no. 6 (2024): bbae489. 10.1093/bib/bbae489 PMC11442149 39350338

[9] D. Hu , K. Liang , Z. Dong , J. Wang , Y. Zhao , and K. He , “Effective Multi‐Modal Clustering Method via Skip Aggregation Network for Parallel scRNA‐Seq and scATAC‐Seq Data,” Briefings in Bioinformatics 25, no. 2 (2024): bbae102. 10.1093/bib/bbae102 PMC10944573 38493338

[10] O. Stegle , S. A. Teichmann , and J. C. Marioni , “Computational and Analytical Challenges in Single‐Cell Transcriptomics,” Nature Reviews Genetics 16, no. 3 (2015): 133–145, 10.1038/nrg3833. 25628217

[11] V. Y. Kiselev , T. S. Andrews , and M. Hemberg , “Challenges in Unsupervised Clustering of Single‐Cell RNA‐Seq Data,” Nature Reviews Genetics 20, no. 5 (2019): 273–282, 10.1038/s41576-018-0088-9. 30617341

[12] J. Wang , J. Xia , H. Wang , Y. Su , and C. H. Zheng , “scDCCA: Deep Contrastive Clustering for Single‐Cell RNA‐Seq Data Based on Auto‐Encoder Network,” Briefings in Bioinformatics 24, no. 1 (2023): bbac625. 10.1093/bib/bbac625 36631401

[13] R. Lopez , J. Regier , M. B. Cole , M. I. Jordan , and N. Yosef , “Deep Generative Modeling for Single‐Cell Transcriptomics,” Nature Methods 15, no. 12 (2018): 1053–1058, 10.1038/s41592-018-0229-2. PMC6289068 30504886

[14] Y. Cheng , X. Fan , J. Zhang , and Y. Li , “A Scalable Sparse Neural Network Framework for Rare Cell Type Annotation of Single‐Cell Transcriptome Data,” Communications Biology 6, no. 1 (2023): 545, 10.1038/s42003-023-04928-6. PMC10199434 37210444

[15] L. Haghverdi , A. T. L. Lun , M. D. Morgan , and J. C. Marioni , “Batch Effects in Single‐Cell RNA‐Sequencing Data are Corrected by Matching Mutual Nearest Neighbors,” Nature Biotechnology 36, no. 5 (2018): 421–427, 10.1038/nbt.4091. PMC6152897 29608177

[16] G. Eraslan , L. M. Simon , M. Mircea , N. S. Mueller , and F. J. Theis , “Single‐Cell RNA‐Seq Denoising Using a Deep Count Autoencoder,” Nature Communications 10, no. 1 (2019): 390, 10.1038/s41467-018-07931-2. PMC6344535 30674886

[17] J. Xie , R. Girshick , and A. Farhadi , “Unsupervised Deep Embedding for Clustering Analysis,” in Proceedings of the 33rd International Conference on Machine Learning, Proceedings of Machine Learning Research (PMLR, 2016), 478–487.

[18] P. Velickovic , W. Fedus , W. L. Hamilton , P. Liò , Y. Bengio , and R. D. Hjelm , “Deep Graph Infomax,” in Proceedings of the 7th International Conference on Learning Representations (ICLR) (2019), 10.48550/arXiv.1809.10341.

[19] W. Tu , S. Zhou , X. Liu , et al., “Deep Fusion Clustering Network,” Proceedings of the AAAI Conference on Artificial Intelligence 35 (2021): 9978–9987.

[20] Z. Jiang , Y. Zheng , H. Tan , B. Tang , and H. Zhou , “Variational Deep Embedding: An Unsupervised and Generative Approach to Clustering,” in Proceedings of the Twenty-Sixth International Joint Conference on Artificial Intelligence (2017): 1965–1972.

[21] S. Mukherjee , H. Asnani , E. Lin , and S. Kannan , “ClusterGAN: Latent Space Clustering in Generative Adversarial Networks,” Proceedings of the AAAI Conference on Artificial Intelligence 33 (2019): 4610–4617, 10.1609/aaai.v33i01.33014610.

[22] X. Ji , J. F. Henriques , and A. Vedaldi , “Invariant Information Clustering for Unsupervised Image Classification and Segmentation,” Proceedings of the IEEE/CVF International Conference on Computer Vision (2019): 9865–9874.

[23] P. Ji , T. Zhang , H. Li , M. Salzmann , and I. Reid , “Deep Subspace Clustering Networks,” Advances in Neural Information Processing Systems 30 (2017): 24–33.

[24] B. Settles , “Active Learning Literature Survey,” Computer Sciences Technical Report 1648, University of Wisconsin‐Madison, Madison, WI (2009).

[25] A. Shrivastava , A. Gupta , and R. Girshick , “Training Region‐Based Object Detectors with Online Hard Example Mining,” in Proceedings of the IEEE Conference on Computer Vision and Pattern Recognition (IEEE, 2016), 761–769.

[26] T.‐Y. Lin , P. Goyal , R. Girshick , K. He , and P. Dollár , “Focal Loss for Dense Object Detection,” in Proceedings of the IEEE International Conference on Computer Vision (IEEE, 2017), 2980–2988.

[27] S. Li , H. Guo , S. Zhang , Y. Li , and M. Li , “Attention‐Based Deep Clustering Method for scRNA‐Seq Cell Type Identification,” PLOS Computational Biology 19, no. 11 (2023): 1011641, 10.1371/journal.pcbi.1011641. PMC10703402 37948464

[28] Y. Zeng , X. Zhou , J. Rao , Y. Lu , and Y. Yang , “Accurately Clustering Single‐Cell RNA‐seq Data by Capturing Structural Relations between Cells Through Graph Convolutional Network,” in 2020 IEEE International Conference on Bioinformatics and Biomedicine (BIBM) (IEEE, 2020), 519–522, 10.1109/BIBM49941.2020.9313569.

[29] Y. Cheng , Y. Su , Z. Yu , Y. Liang , K.‐C. Wong , and X. Li , “Unsupervised Deep Embedded Fusion Representation of Single‐Cell Transcriptomics,” Proceedings of the AAAI Conference on Artificial Intelligence 37 (2023): 5036–5044, 10.1609/aaai.v37i4.25631.

[30] T. Tian , J. Wan , Q. Song , and Z. Wei , “Clustering Single‐Cell RNA‐seq Data With a Model‐Based Deep Learning Approach,” Nature Machine Intelligence 1, no. 4 (2019): 191–198, 10.1038/s42256-019-0037-0.

[31] Y. Qiu , L. Yang , H. Jiang , and Q. Zou , “scTPC: A Novel Semisupervised Deep Clustering Model for scRNA‐seq Data,” Bioinformatics 40, no. 5 (2024): btae293, 10.1093/bioinformatics/btae293. PMC11091743 38684178

[32] Y. Qiu , C. Yan , P. Zhao , and Q. Zou , “SSNMDI: A Novel Joint Learning Model of Semi‐Supervised Non‐Negative Matrix Factorization and Data Imputation for Clustering of Single‐Cell RNA‐seq Data,”Briefings in Bioinformatics 24, no. 3 (2023): bbad149. 10.1093/bib/bbad149 37122068

[33] L. Hubert and P. Arabie , “Comparing Partitions,” Journal of Classification 2 (1985): 193–218, 10.1007/BF01908075.

[34] A. Strehl and J. Ghosh , “Cluster Ensembles—A Knowledge Reuse Framework for Combining Multiple Partitions,” Journal of Machine Learning Research 3 (2002): 583–617.

[35] S. Zhang , Y. Cui , X. Ma , et al., “Single‐Cell Transcriptomics Identifies Divergent Developmental Lineage Trajectories During Human Pituitary Development,” Nature Communications 11, no. 1 (2020): 5275, 10.1038/s41467-020-19012-4. PMC7572359 33077725

[36] S. Jin , C. F. Guerrero‐Juarez , L. Zhang , et al., “Inference and Analysis of Cell‐Cell Communication using CellChat,” Nature Communications 12, no. 1 (2021): 1088, 10.1038/s41467-021-21246-9. PMC7889871 33597522

[37] P. Zhou , S. Wang , T. Li , and Q. Nie , “Dissecting Transition Cells From Single‐Cell Transcriptome Data Through Multiscale Stochastic Dynamics,” Nature Communications 12, no. 1 (2021): 5609, 10.1038/s41467-021-25548-w. PMC8460805 34556644

[38] S. Mohammadi , J. Davila‐Velderrain , and M. Kellis , “A Multiresolution Framework to Characterize Single‐Cell State Landscapes,” Nature Communications 11, no. 1 (2020): 5399, 10.1038/s41467-020-18416-6. PMC7588427 33106496

[39] S. W. Davis , A. H. Mortensen , J. L. Keisler , et al., “β‐Catenin is Required in the Neural Crest and Mesencephalon for Pituitary Gland Organogenesis,” BMC Developmental Biology 16, no. 1 (2016): 16, 10.1186/s12861-016-0118-9. PMC4868042 27184910

[40] S. Artavanis‐Tsakonas , M. D. Rand , and R. J. Lake , “Notch Signaling: Cell Fate Control and Signal Integration in Development,” Science 284, no. 5415 (1999): 770–776, 10.1126/science.284.5415.770. 10221902

[41] R. O. Hynes , “The Extracellular Matrix: Not Just Pretty Fibrils,” Science 326, no. 5957 (2009): 1216–1219, 10.1126/science.1176009. PMC3536535 19965464

[42] T. Rozario and D. W. DeSimone , “The Extracellular Matrix in Development and Morphogenesis: A Dynamic View,” Developmental Biology 341, no. 1 (2010): 126–140, 10.1016/j.ydbio.2009.10.026. PMC2854274 19854168

[43] J. Andrae , R. Gallini , and C. Betsholtz , “Role of Platelet‐Derived Growth Factors in Physiology and Medicine,” Genes & Development 22, no. 10 (2008): 1276–1312, 10.1101/gad.1653708. PMC2732412 18483217

[44] H. Clevers and R. Nusse , “Wnt/β‐Catenin Signaling and Disease,” Cell 149, no. 6 (2012): 1192–1205, 10.1016/j.cell.2012.05.012. 22682243

[45] C. Gaston‐Massuet , C. L. Andoniadou , M. Signore , et al., “Increased Wingless ( Wnt ) Signaling in Pituitary Progenitor/Stem Cells Gives Rise to Pituitary Tumors in Mice and Humans,” Proceedings of the National Academy of Sciences 108, no. 28 (2011): 11482–11487, 10.1073/pnas.1101553108. PMC3136310 21636786

[46] S. Hrvatin , D. R. Hochbaum , M. A. Nagy , et al., “Single‐Cell Analysis of Experience‐Dependent Transcriptomic States in the Mouse Visual Cortex,” Nature Neuroscience 21, no. 1 (2018): 120–129, 10.1038/s41593-017-0029-5. PMC5742025 29230054

[47] A. M. Bell , C. Utting , A. C. Dickie , et al., “Deep Sequencing of Phox2a Nuclei Reveals Five Classes of Anterolateral System Neurons,” Proceedings of the National Academy of Sciences 121, no. 23 (2024): 2314213121, 10.1073/pnas.2314213121. PMC11161781 38805282

[48] D. C. Schwalbe , D. S. Stornetta , R. J. Abraham‐Fan , et al., “Molecular Organization of Autonomic, Respiratory, and Spinally‐Projecting Neurons in the Mouse Ventrolateral Medulla,” Journal of Neuroscience 44, no. 31 (2024): 2211232024. 10.1523/JNEUROSCI.2211-23.2024 PMC11293450 38918066

[49] F. Scala , D. Kobak , M. Bernabucci , et al., “Phenotypic Variation of Transcriptomic Cell Types in Mouse Motor Cortex,” Nature 598, no. 7879 (2021): 144–150, 10.1038/s41586-020-2907-3. PMC8113357 33184512

[50] P. Savas , B. Virassamy , C. Ye , et al., “Single‐Cell Profiling of Breast Cancer T Cells Reveals a Tissue‐Resident Memory Subset Associated With Improved Prognosis,” Nature Medicine 24, no. 7 (2018): 986–993, 10.1038/s41591-018-0078-7. 29942092

[51] R. Satija , J. A. Farrell , D. Gennert , A. F. Schier , and A. Regev , “Spatial Reconstruction of Single‐Cell Gene Expression Data,” Nature Biotechnology 33, no. 5 (2015): 495–502, 10.1038/nbt.3192. PMC4430369 25867923

[52] P. Bankhead , M. B. Loughrey , J. A. Fernández , et al., “QuPath: Open Source Software for Digital Pathology Image Analysis,” Scientific Reports 7, no. 1 (2017): 16878, 10.1038/s41598-017-17204-5. PMC5715110 29203879

[53] J. M. Courtney , G. P. Morris , E. M. Cleary , D. W. Howells , and B. A. Sutherland , “An Automated Approach to Improve the Quantification of Pericytes and Microglia in Whole Mouse Brain Sections,” eNeuro 8, no. 6 (2021): ENEURO.0177–21.2021. 10.1523/ENEURO.0177-21.2021 PMC8570687 34642225

[54] F. A. Wolf , P. Angerer , and F. J. Theis , “SCANPY: Large‐Scale Single‐Cell Gene Expression Data Analysis,” Genome Biology 19, no. 1 (2018): 15, 10.1186/s13059-017-1382-0. PMC5802054 29409532

