# Supplementary material for: scGEN: Leveraging an Adaptive Weighting Strategy for Challenging Cell Clustering in Single‐Cell RNA‐seq
Source: Adv Sci (Weinh). 2026 Oct 11:e77746. Online ahead of print. doi: 10.1002/advs.77746 (PMC13655258; doi:10.1002/advs.77746)
Supplement: Supplementary file 1 [file ADVS-9999-e77746-s001.docx]

Supporting Information

**scGEN: Leveraging an Adaptive Weighting Strategy for Challenging Cell Clustering in Single-cell RNA-seq**

*Fang Huang^1,2,3,*^, Dayu Hu^4,*^, Yaqi Dai^1,2,*^, Aoyi Geng^1,2^, Renxiang Guan^4^, Hao Quan^4^, Brett A. McGregor^3^, Zhihan Wang^5^, Nadeem Khan^6^, Hongsheng Zhang^1,2^, Jinghua Zhang^7^, Mingyang Xia^8,9^ ,* *Xiaoqiong Gu^10,11^,* *Di Che^10^, Junguk Hur^3,†^, Xiaoyu Cui^4,†^, and Kai Guo^3,12,†^*

^1^Department of Neurobiology, School of Basic Medical Sciences, Chongqing Medical University, Chongqing 400016, China

^2^Key Laboratory of Major Brain Disease and Aging Research (Ministry of Education), Chongqing Medical University, Chongqing, 400016, China

^3^Department of Biomedical Sciences, University of North Dakota, School of Medicine and Health Sciences, Grand Forks, ND 58202, USA

^4^College of Medicine and Biological Information Engineering, Northeastern University, Shenyang, 110169, China

^5^Department of Human Anatomy, School of Basic Medical Sciences, Chengdu Medical College, Chengdu, 610500, China

^6^Department of Oral Biology, College of Dentistry, University of Florida, Gainesville, FL 32610, USA

^7^College of Computer Science and Software Engineering, Hohai University, Nanjing, 21100, China

^8^Institute of Pediatrics, Guangzhou Women and Children's Medical Center, Guangzhou Medical University, Guangzhou, China

^9^Key Laboratory of Pediatric Rare Diseases of Guangdong Higher Education Institutes, Guangzhou Medical University, Guangzhou, 510623, China

^10^Department of Clinical Biological Resource Bank, Guangzhou Women and Children’s Medical Center, Guangzhou Medical University, Guangzhou, 510623, China

^11^Department of Molecular Medical Center, Guangzhou Women and Children's Medical Center, Guangzhou Medical University, Guangzhou, 510623, China

^12^Department of Neurology, University of Michigan, Ann Arbor, MI 48109, USA

* These authors contributed equally to this study and share the first authorship.

**This Supporting Information includes:**

Supplementary Figures S1-S5

Supplementary Tables S1-S8

**Supplementary Figures**

**Figure S1.** scGEN reveals inconsistencies in cell-type annotations in human fetal pituitary single-cell RNA-seq data. (A) Feature plots showing gene expression of key marker genes across the pituitary cell population. Each plot displays the expression pattern of an individual gene (PDGFRA, CDH5, TBX19, EPB42, TSHB, PRL, SLITRK1, SOX2, GH2, MKI67, CORO1A, and GNRHR) projected onto UMAP coordinates. (B) UMAP visualization of cell clustering results from seven different methods (GraphSCC, scDEFR, scDeepCluster, scDCCA, K-means, Spectral and scTPC) compared with the original annotations. Each cluster is color-coded according to the cell type legend showing 14 distinct pituitary cell populations including Corticotrope, MC, Stem, CC, PP, Imm, Pro.PIT1, EC, RBC, Pre.Gonado, Somatotrope, Thyrotrope, Gonadotrope, and Lactotrope. (C) Comparison of consistently and inconsistently annotated cells across clustering methods. UMAP plots highlight in red the cells that were inconsistently annotated across GraphSCC, scDEFR, scDeepCluster, scDCCA, K-means, Spectral, scTPC, and scGEN methods compared to the original annotations. Gray cells indicate consistently annotated cells. (D) Box plots comparing cell type scores between consistent and inconsistent cell annotations across four clustering methods (GraphSCC, scDEFR, scDeepCluster, and K-means) for different cell lineages (MC, PP, PIT1 lineage, Gonado lineage, and CC).

**Figure S2.** Functional enrichment analysis with Gene Ontology (GO) for the shared 48 DEGs, color stands for the up-regulated genes (A) or down-regulated genes (B) were used as input.

**Figure S3. Gonadotrope-lineage module scores across three groups.** Gonadotrope-lineage module scores. Statistical significance indicated by asterisks: ****p<0.0001, ns=not significant.

**Figure S4. scGEN uncovers enhanced growth factor and developmental signaling networks involving Pre.Gonado cells.** **(A)** Chord diagrams showing differences in PDGF and WNT signaling pathways between original and scGEN annotations. Dark-orange links represent increased activity in scGEN, while blue links represent stronger signaling in original annotations. Pre.Gonado cells show enhanced integration into growth factor and morphogen pathways under scGEN refinement. **(B)** Representative network views of PDGF and SLITRK signaling pathways at 7 weeks. Nodes are colored by cell type. **(C)** Ligand-receptor analysis of classical developmental pathways (BMP, NOTCH, SLITRK) showing outgoing signals from Pre.Gonado cells to other cell types at 7 weeks. Color indicates communication probability and dot size indicates statistical significance, Wilcoxon test. **(D)** Ligand-receptor interaction map detailing BMP, NOTCH, and SLIT pathways showing incoming signals to Pre.Gonado cells from other cell types at 7 weeks. MC, mesenchymal cells, EC, endothelial cells, Imm, immune cells, PP, pituicytes, CC, cycling cells, RBC, red blood cells.

**Figure S5. Double-positive bridging populations during the progenitor-to-gonadotrope transition.** Quantification of SOX2⁺GATA2⁺ and GATA2⁺NR5A1⁺ cells across 6-8 week embryos in the Rathke’s pouch/pituitary domain. Percentages were calculated relative to total DAPI-detected cells per ROI; data represent individual values and mean ± SD from n = 8 ROIs per week. One-way ANOVA followed by Tukey’s post-hoc tests determined significance. SOX2⁺GATA2⁺ cells decrease over development, whereas GATA2⁺NR5A1⁺ cells increase, consistent with transitions through Pre.Gonado.

**Supplementary Tables**

**Table S1. Quantitative clustering performance comparison across eight scRNA-seq datasets, measured by ACC, NMI and ARI. The best scores in each metric in each dataset are highlighted in bold.**

| **Dataset** | **Metric** | **AttentionAE-sc** | **GraphSCC** | **kmeans** | **scDCCA** | **scDEFR** | **scDeepCluster** | **scTPC** | **Spectral clustering** | **SSNMDI** | **scGEN** |
| --- | --- | --- | --- | --- | --- | --- | --- | --- | --- | --- | --- |
| Bell | ACC | 40.29 ± 13.73 | 81.31 ± 9.26 | 47.33 ± 0.04 | 54.37 ± 0.00 | 89.08 ± 1.03 | 76.94 ± 1.71 | 82.77 ± 0.02 | 60.68 ± 0.00 | 66.87 ± 0.02 | **90.29 ± 0.00** |
|  | NMI | 57.97 ± 2.40 | 69.28 ± 4.96 | 45.00 ± 0.04 | 45.61 ± 0.00 | 74.71 ± 2.01 | 67.20 ± 0.08 | 77.27 ± 0.01 | 42.04 ± 0.00 | 63.95 ± 0.01 | **77.81 ± 0.00** |
|  | ARI | 45.85 ± 1.16 | 64.83 ± 9.73 | 32.57 ± 0.03 | 36.21 ± 0.00 | 74.27 ± 2.31 | 60.10 ± 0.91 | 73.63 ± 0.00 | 23.65 ± 0.00 | 54.05 ± 0.02 | **77.14 ± 0.00** |
| Schwalbe | ACC | 14.82 ± 4.51 | 38.76 ± 3.08 | 35.96 ± 0.04 | 38.48 ± 0.00 | 47.49 ± 0.71 | 38.87 ± 2.45 | 46.36 ± 0.03 | 44.46 ± 0.00 | 50.77 ± 0.05 | **56.54 ± 1.17** |
|  | NMI | 6.92 ± 0.83 | 36.12 ± 4.19 | 30.11 ± 0.08 | 25.68 ± 0.00 | 44.54 ± 0.49 | 32.74 ± 3.10 | **57.59 ± 0.01** | 43.96 ± 0.00 | 45.81 ± 0.04 | 48.07 ± 0.77 |
|  | ARI | 2.24 ± 1.37 | 20.11 ± 3.88 | 13.92 ± 0.09 | 15.42 ± 0.00 | 26.62 ± 0.31 | 17.45 ± 2.63 | **38.35 ± 0.04** | 25.56 ± 0.00 | 30.02 ± 0.04 | 29.12 ± 0.27 |
| Scala | ACC | 7.86 ± 1.75 | 29.69 ± 2.07 | 44.58 ± 0.10 | 35.21 ± 0.00 | 57.90 ± 0.16 | 37.10 ± 2.45 | 65.27 ± 0.01 | 55.30 ± 0.00 | 58.83 ± 0.04 | **69.68 ± 0.83** |
|  | NMI | 14.46 ± 5.27 | 14.25 ± 5.13 | 35.55 ± 0.10 | 30.35 ± 0.00 | 56.38 ± 0.33 | 33.52 ± 0.19 | **71.50 ± 0.07** | 51.06 ± 0.00 | 51.19 ± 0.01 | 62.76 ± 0.53 |
|  | ARI | 7.90 ± 3.37 | 7.45 ± 3.21 | 28.75 ± 0.10 | 18.19 ± 0.00 | 44.16 ± 0.19 | 21.55 ± 0.21 | 57.01 ± 0.06 | 37.87 ± 0.00 | 45.19 ± 0.05 | **58.59 ± 1.07** |
| Hrvatin B1 | ACC | 12.94 ± 1.50 | 92.90 ± 1.18 | 83.38 ± 0.03 | 91.28 ± 0.00 | 87.29 ± 4.76 | 90.80 ± 1.32 | 91.01 ± 0.05 | 88.66 ± 0.00 | 83.93 ± 0.00 | **96.41 ± 0.69** |
|  | NMI | 90.87 ± 1.99 | 93.13 ± 0.81 | 88.17 ± 0.00 | 92.75 ± 0.00 | 91.17 ± 1.86 | 91.60 ± 0.63 | 91.32 ± 0.02 | 83.42 ± 0.00 | 90.04 ± 0.00 | **96.22 ± 0.59** |
|  | ARI | 88.84 ± 2.02 | 92.31 ± 1.38 | 81.96 ± 0.04 | 90.30 ± 0.00 | 88.67 ± 3.61 | 89.38 ± 2.42 | 91.35 ± 0.04 | 79.30 ± 0.00 | 86.70 ± 0.00 | **96.32 ± 0.13** |
| PBMC 3K | ACC | 3.49 ± 3.27 | 68.44 ± 2.60 | 76.40 ± 0.02 | 66.53 ± 0.00 | 74.70 ± 0.08 | 69.47 ± 0.19 | 80.97 ± 0.04 | 69.14 ± 0.00 | 83.62 ± 0.02 | **86.03 ± 0.06** |
|  | NMI | 46.00 ± 2.38 | 78.42 ± 0.71 | 76.69 ± 0.01 | 75.90 ± 0.00 | **80.24 ± 0.19** | 77.35 ± 0.38 | 75.19 ± 0.09 | 72.64 ± 0.00 | 77.50 ± 0.01 | 79.31 ± 0.01 |
|  | ARI | 22.53 ± 0.45 | 61.32 ± 1.08 | 65.55 ± 0.02 | 60.74 ± 0.00 | 66.34 ± 0.14 | 60.54 ± 0.54 | **71.61 ± 0.11** | 56.27 ± 0.00 | 66.33 ± 0.03 | 69.39 ± 0.18 |
| Hrvatin B2 | ACC | 19.94 ± 0.26 | 86.52 ± 0.61 | 84.46 ± 0.04 | 88.88 ± 0.00 | 81.28 ± 1.04 | 85.84 ± 0.86 | 83.08 ± 0.03 | 84.30 ± 0.00 | 88.06 ± 0.01 | **93.09 ± 0.23** |
|  | NMI | 90.40 ± 0.22 | 90.88 ± 0.18 | 85.45 ± 0.05 | 91.66 ± 0.00 | 79.41 ± 2.73 | 87.13 ± 1.19 | 86.08 ± 0.04 | 78.57 ± 0.00 | 88.76 ± 0.00 | **93.23 ± 1.76** |
|  | ARI | 83.52 ± 0.39 | 83.88 ± 0.42 | 75.01 ± 0.09 | 85.22 ± 0.00 | 69.38 ± 6.12 | 80.37 ± 0.44 | 82.78 ± 0.04 | 67.86 ± 0.00 | 82.68 ± 0.01 | **88.95 ± 1.16** |
| Zhang | ACC | 3.24 ± 1.48 | 70.14 ± 1.51 | 65.22 ± 0.07 | 70.02 ± 0.00 | 81.04 ± 8.36 | 76.47 ± 1.77 | 83.31 ± 0.12 | 77.75 ± 0.00 | 71.06 ± 0.09 | **90.64 ± 0.39** |
|  | NMI | 28.15 ± 39.81 | 79.03 ± 0.59 | 76.92 ± 0.04 | 76.45 ± 0.00 | **87.41 ± 5.08** | 81.45 ± 0.81 | 85.31 ± 0.01 | 79.66 ± 0.00 | 76.71 ± 0.05 | 85.74 ± 0.36 |
|  | ARI | 14.97 ± 21.17 | 67.10 ± 0.80 | 60.06 ± 0.09 | 65.82 ± 0.00 | 78.59 ± 7.91 | 69.75 ± 0.16 | 86.60 ± 0.04 | 75.78 ± 0.00 | 68.95 ± 0.13 | **90.03 ± 0.41** |
| Savas | ACC | 4.29 ± 2.06 | 65.58 ± 5.71 | 52.06 ± 0.02 | 66.80 ± 0.00 | 75.86 ± 0.01 | 65.02 ± 0.78 | 68.81 ± 0.12 | 63.87 ± 0.00 | 61.85 ± 0.03 | **77.83 ± 0.43** |
|  | NMI | 18.12 ± 19.58 | 56.98 ± 1.57 | 47.69 ± 0.01 | 52.53 ± 0.00 | **61.38 ± 0.14** | 57.61 ± 2.21 | 55.09 ± 0.13 | 54.00 ± 0.00 | 51.75 ± 0.01 | 60.79 ± 0.47 |
|  | ARI | 11.12 ± 12.94 | 49.98 ± 4.95 | 36.52 ± 0.00 | 52.92 ± 0.00 | 61.40 ± 0.28 | 49.94 ± 0.87 | 60.84 ± 0.18 | 43.74 ± 0.00 | 44.11 ± 0.01 | **62.87 ± 0.37** |

**Table S2. The clustering results of the ablation study. The best scores in each metric in each dataset are highlighted in bold.**

| **Dataset** | **Metric** | **scGEN w/o ZINB** | **scGEN w/o HSL** | **scGEN** |
| --- | --- | --- | --- | --- |
| Bell | ACC | 88.83 ± 0.00 | 89.32 ± 0.00 | **90.29 ± 0.00** |
|  | NMI | 74.65 ± 0.00 | 75.68 ± 0.21 | **77.81 ± 0.00** |
|  | ARI | 74.02 ± 0.00 | 75.09 ± 0.06 | **77.14 ± 0.00** |
| Schwalbe | ACC | 52.18 ± 2.07 | 55.82 ± 2.31 | **56.54 ± 1.17** |
|  | NMI | 44.94 ± 0.01 | **48.75 ± 1.25** | 48.07 ± 0.77 |
|  | ARI | 28.04 ± 0.17 | **29.20 ± 1.41** | 29.12 ± 0.27 |
| Scala | ACC | 66.59 ± 1.05 | 58.77 ± 0.30 | **69.68 ± 0.83** |
|  | NMI | 60.15 ± 1.75 | 56.00 ± 2.56 | **62.76 ± 0.53** |
|  | ARI | 55.08 ± 1.77 | 42.66 ± 2.27 | **58.59 ± 1.07** |
| Hrvatin B1 | ACC | 81.06 ± 1.25 | 88.14 ± 0.31 | **96.41 ± 0.69** |
|  | NMI | 87.92 ± 1.18 | 90.33 ± 0.32 | **96.22 ± 0.59** |
|  | ARI | 81.18 ± 0.83 | 87.78 ± 0.80 | **96.32 ± 0.13** |
| PBMC 3K | ACC | 82.56 ± 2.35 | 78.28 ± 0.76 | **86.03 ± 0.06** |
|  | NMI | 77.66 ± 0.69 | 76.07 ± 0.45 | **79.31 ± 0.01** |
|  | ARI | 65.17 ± 2.10 | 64.64 ± 0.06 | **69.39 ± 0.18** |
| Hrvatin B2 | ACC | 85.87 ± 4.73 | 85.82 ± 2.03 | **93.09 ± 0.23** |
|  | NMI | 89.19 ± 2.98 | 84.44 ± 0.93 | **93.23 ± 1.76** |
|  | ARI | 83.19 ± 5.61 | 77.92 ± 2.43 | **88.95 ± 1.16** |
| Zhang | ACC | 68.25 ± 0.88 | 75.59 ± 8.12 | **90.64 ± 0.39** |
|  | NMI | 76.35 ± 1.45 | 75.03 ± 7.27 | **85.74 ± 0.36** |
|  | ARI | 66.00 ± 6.92 | 67.59 ± 16.10 | **90.03 ± 0.41** |
| Savas | ACC | 68.21 ± 1.07 | 67.62 ± 0.64 | **77.83 ± 0.43** |
|  | NMI | 56.67 ± 0.53 | 54.85 ± 0.40 | **60.79 ± 0.47** |
|  | ARI | 55.47 ± 0.76 | 54.50 ± 1.26 | **62.87 ± 0.37** |

**Table S3. Quantitative clustering performance comparison of different resolutions used by Seurat v5 across eight scRNA-seq datasets, measured by ACC, NMI, and ARI. The scores better than scGEN are highlighted in bold.**

| **Dataset** | **Resolution** | **Seurat version used in paper** | **#clusters in paper** | **#Clusters by Seurat v5** | **ACC** | **NMI** | **ARI** |
| --- | --- | --- | --- | --- | --- | --- | --- |
| Bell | 0.9 | 4.1.4 | 5 | 3 | 61.65 | 46.08 | 46.29 |
|  | 1 |  |  | 3 | 62.14 | 49.84 | 50.01 |
|  | 1.1 |  |  | 4 | 66.50 | 50.74 | 49.19 |
|  | 1.2 |  |  | 6 | 80.58 | 63.89 | 61.25 |
|  | 1.3 |  |  | 9 | 77.18 | 60.93 | 58.90 |
| Schwalbe | 0.5 | 4.1.1 | 9 | 7 | 55.15 | **53.29** | **37.21** |
|  | 0.6 |  |  | 8 | 53.13 | **53.01** | **34.87** |
|  | 0.7 |  |  | 9 | **57.94** | **58.07** | **39.50** |
|  | 0.8 |  |  | 10 | 50.00 | **58.16** | **39.32** |
|  | 0.9 |  |  | 11 | 50.00 | **57.98** | **41.38** |
| Scala | 1.6 | - | 10 | 8 | 65.61 | 56.66 | 52.50 |
|  | 1.7 |  |  | 9 | 63.21 | 55.74 | 50.85 |
|  | 1.8 |  |  | 10 | 62.60 | 54.91 | 52.14 |
|  | 1.9 |  |  | 10 | 60.95 | 54.84 | 51.16 |
|  | 2 |  |  | 12 | 59.29 | 53.39 | 49.32 |
| Hrvatin B1 | 0.1 | Not specified | 8 | 9 | 84.13 | 84.81 | 83.69 |
|  | 0.2 |  |  | 10 | 80.35 | 82.05 | 81.49 |
|  | 0.3 |  |  | 13 | 73.16 | 77.26 | 78.54 |
|  | 0.4 |  |  | 13 | 73.16 | 77.26 | 78.54 |
|  | 0.5 |  |  | 15 | 69.63 | 73.12 | 74.71 |
| PBMC 3K | 0.7 | 4.1.4 | 9 | 9 | **92.15** | **85.47** | **80.63** |
|  | 0.8 |  |  | 9 | **92.15** | **85.47** | **80.63** |
|  | 0.9 |  |  | 9 | **92.15** | **85.53** | **80.62** |
|  | 1 |  |  | 9 | **92.19** | **85.59** | **80.70** |
|  | 1.1 |  |  | 9 | **91.89** | **85.31** | **80.00** |
| Hrvatin B2 | 0.1 | Not specified | 8 | 12 | 75.33 | 75.54 | 70.94 |
|  | 0.2 |  |  | 14 | 66.24 | 69.93 | 62.95 |
|  | 0.3 |  |  | 17 | 63.01 | 66.12 | 58.83 |
|  | 0.4 |  |  | 18 | 59.20 | 63.82 | 54.21 |
|  | 0.5 |  |  | 19 | 58.03 | 62.52 | 52.71 |
| Zhang | 0.1 | 2.3.4 | 14 | 12 | 82.40 | 81.39 | 80.99 |
|  | 0.2 |  |  | 12 | 81.81 | 80.96 | 80.31 |
|  | 0.3 |  |  | 12 | 81.79 | 81.43 | 80.88 |
|  | 0.4 |  |  | 15 | 74.18 | 79.41 | 69.25 |
|  | 0.5 |  |  | 17 | 77.95 | 78.68 | 71.86 |
| Savas | 0.1 | 4.1.3 | 10 | 6 | 64.25 | 47.01 | 47.39 |
|  | 0.2 |  |  | 8 | 68.10 | 53.46 | 54.22 |
|  | 0.3 |  |  | 10 | **80.73** | **62.07** | **64.61** |
|  | 0.4 |  |  | 10 | **80.85** | **62.20** | **64.82** |
|  | 0.5 |  |  | 11 | 74.42 | 58.38 | 60.28 |

**Table S4. Quantitative clustering performance comparison of different resolutions used by Scanpy across eight scRNA-seq datasets, measured by ACC, NMI and ARI. The scores better than scGEN are highlighted in bold.**

| **Dataset** | **resolution** | **raw clusters** | **new clusters** | **ACC** | **NMI** | **ARI** |
| --- | --- | --- | --- | --- | --- | --- |
| Bell | 0.7 | 5 | 5 | 88.83 | 72.85 | 72.65 |
|  | 0.8 |  | 5 | 88.35 | 72.89 | 71.59 |
|  | 0.9 |  | 5 | 88.35 | 71.50 | 71.69 |
|  | 1 |  | 5 | 88.83 | 73.14 | 73.25 |
|  | 1.1 |  | 5 | 88.35 | 71.51 | 71.93 |
| Schwalbe | 0.6 | 9 | 6 | 43.74 | 38.87 | 23.71 |
|  | 0.7 |  | 8 | 43.40 | 44.75 | 27.51 |
|  | 0.8 |  | 9 | 46.20 | 46.26 | 28.82 |
|  | 0.9 |  | 11 | 44.52 | 47.69 | **29.90** |
|  | 1 |  | 12 | 43.40 | **48.27** | **29.44** |
| Scala | 0.4 | 10 | 9 | 62.98 | 60.38 | 47.25 |
|  | 0.5 |  | 9 | 62.98 | 60.38 | 47.25 |
|  | 0.6 |  | 11 | 66.22 | 62.10 | 48.70 |
|  | 0.7 |  | 11 | 62.23 | 61.18 | 48.40 |
|  | 0.8 |  | 12 | 63.66 | **64.05** | 52.25 |
| Hrvatin B1 | 0.1 | 8 | 7 | 90.69 | 93.42 | 91.53 |
|  | 0.2 |  | 9 | 92.15 | 93.66 | 91.99 |
|  | 0.3 |  | 10 | 93.27 | 94.68 | 92.54 |
|  | 0.4 |  | 11 | 90.82 | 93.06 | 90.47 |
|  | 0.5 |  | 12 | 86.41 | 90.31 | 84.77 |
| PBMC 3K | 1 | 9 | 8 | 80.78 | 76.37 | 66.38 |
|  | 1.1 |  | 8 | 79.76 | 75.96 | 64.37 |
|  | 1.2 |  | 8 | 77.52 | 75.19 | 61.28 |
|  | 1.3 |  | 9 | 82.56 | 77.23 | 64.13 |
|  | 1.4 |  | 10 | 74.72 | 73.26 | 59.52 |
| Hrvatin B2 | 0.1 | 8 | 10 | 88.94 | 91.20 | 84.52 |
|  | 0.2 |  | 11 | 82.37 | 87.84 | 75.29 |
|  | 0.3 |  | 12 | 81.47 | 87.07 | 74.37 |
|  | 0.4 |  | 13 | 79.35 | 85.58 | 71.55 |
|  | 0.5 |  | 15 | 77.39 | 84.57 | 69.01 |
| Zhang | 0.1 | 14 | 10 | 70.14 | 75.83 | 63.03 |
|  | 0.2 |  | 13 | 81.55 | 82.85 | 83.11 |
|  | 0.3 |  | 14 | 80.23 | 81.97 | 80.13 |
|  | 0.4 |  | 15 | 80.84 | 82.17 | 79.99 |
|  | 0.5 |  | 17 | 76.32 | 80.74 | 70.20 |
| Savas | 0.4 | 10 | 9 | 77.77 | **61.67** | 62.02 |
|  | 0.5 |  | 9 | **77.95** | **61.73** | 62.32 |
|  | 0.6 |  | 10 | 73.24 | 59.56 | 58.86 |
|  | 0.7 |  | 11 | 74.51 | 59.86 | 59.36 |
|  | 0.8 |  | 11 | 74.89 | 60.21 | 59.98 |

**Table S5. Description of notations used in this study.**

| **Notation** | **Definition** |
| --- | --- |
| $X$ | The original single-cell input matrix. |
| $N$ | The number of cells. |
| $D$ | The feature dimension of the cell matrix. |
| V | The nodes of the graph. |
| E | The edges of the graph. |
| $A$ | The constructed adjacency matrix. |
| $\mathcal{F}_{\mathcal{a}}$ | The attribute encoder. |
| $\mathcal{F}_{s}$ | The structure encoder. |
| $H_{A}$ | The attribute embeddings. |
| $H_{s}$ | The structure embeddings. |
| $\mathcal{S}_{\mathcal{ATT}}$ | The attribute similarity. |
| $\mathcal{S}_{\mathcal{STR}}$ | The structural similarity. |
| $\mathcal{S}_{\mathcal{J}}$ | The constructed attribute-structural joint similarity. |
| *β* | The parameter balancing the contributions of attribute and structural information. |
| $H_{\mathcal{J}}$ | The constructed attribute-structural joint embedding. |
| **P** | The pseudo-labels for each cell. |
| $\mathbf{R}$ | The reliable samples. |
| $\mathbf{Q}$ | The sample pairs. |
| $\mathcal{M}$ | The sample pair weight. |
| $\mathcal{L}_{\mathcal{H}}$ | The structure-guided hard-sample contrastive loss. |
| $\mathcal{L}_{\mathcal{Z}}$ | The improved ZINB loss. |
| $t$ | The training epochs. |
| $\Phi$ | The clustering results. |
| $\alpha$ | The trade-off parameter. |
| $\gamma$ | The attention weight parameter. |
| $M$ | The mean of data distribution. |
| $\Theta$ | The dispersion of data distribution. |
| $\Pi$ | The dropout probability of data distribution. |

**Table S6. Description of the eight datasets used in this experiment.**

| **Dataset** | **Gene number** | **Sample size** | **Cluster number** | **Species** | **Platform** |
| --- | --- | --- | --- | --- | --- |
| Bell | 35,429 | 206 | 5 | Mouse | Smart-seq2 |
| Schwalbe | 32,285 | 894 | 9 | Mouse | 10x Genomics Chromium |
| Scala | 42,466 | 1,329 | 10 | Mouse | Smart-seq2 |
| Hrvatin B1 | 17,018 | 2,407 | 8 | Mouse | inDrops |
| PBMC 3K | 13,714 | 2,638 | 9 | Human | 10x Genomics Chromium |
| Hrvatin B2 | 17,509 | 3,012 | 8 | Mouse | inDrops |
| Zhang | 22,610 | 4,113 | 14 | Human | STRT-seq |
| Savas | 15,623 | 5,759 | 10 | Human | 10x Genomics Chromium |

**Table S7. A summary of baseline clustering methods.**

| **Tool** | **Year** | **Model** | **Autoencoder** |
| --- | --- | --- | --- |
| AttentionAE-sc | 2023 | Attention, autoencoder | Denoising & GNN-based autoencoder |
| GraphSCC | 2020 | Graph convolutional network, autoencoder | Denoising autoencoder |
| scDEFR | 2023 | Deep embedded fusion, autoencoder | Deep & graph autoencoder |
| scDeepCluster | 2019 | Auto deep embedded clustering, encoder | Denoising autoencoder |
| scDCC | 2023 | Autoencoder, contrastive learning | Denoising autoencoder |
| k-means | - | Distance-based clustering algorithm | None |
| Spectral Clustering | - | Sample similarity-based clustering algorithm | None |
| SSNMDI | 2023 | Semi-supervised non-negative matrix factorization with imputation | None |
| scTPC | 2024 | Semi-supervised deep clustering with ZINB autoencoder, triplet, pairwise, and cross-entropy constraints | ZINB-based denoising autoencoder |

**Table S8. Sensitivity analysis of the HVG weighting ratio across eight scRNA-seq datasets. The 10% ratio was used as a fixed default rather than selected as a dataset-specific optimum. No single ratio was consistently optimal across all datasets and metrics.**

| Datasets | Metric | 5% | 10% | 15% | 20% | 25% | 30% |
| --- | --- | --- | --- | --- | --- | --- | --- |
| Bell | ACC | **90.29 ± 0.00** | **90.29 ± 0.00** | 90.05 ± 0.34 | 89.81 ± 0.00 | 89.81 ± 0.00 | 89.81 ± 0.00 |
| Bell | NMI | **77.81 ± 0.00** | **77.81 ± 0.00** | 77.12 ± 0.98 | 76.43 ± 0.00 | 76.43 ± 0.00 | 76.43 ± 0.00 |
| Bell | ARI | **77.14 ± 0.00** | **77.14 ± 0.00** | 76.61 ± 0.76 | 76.07 ± 0.00 | 76.07 ± 0.00 | 76.07 ± 0.00 |
| Schwalbe | ACC | 56.82 ± 1.23 | 56.54 ± 1.17 | 56.32 ± 1.29 | 56.27 ± 1.35 | 56.60 ± 0.95 | **56.94 ± 0.71** |
| Schwalbe | NMI | **49.23 ± 0.21** | 48.07 ± 0.77 | 47.36 ± 1.23 | 48.63 ± 0.05 | 47.59 ± 0.97 | 48.47 ± 0.35 |
| Schwalbe | ARI | **29.56 ± 0.36** | 29.12 ± 0.27 | 28.27 ± 0.47 | 29.01 ± 0.28 | 28.38 ± 0.49 | 28.56 ± 0.36 |
| Scala | ACC | 68.97 ± 0.72 | **69.68 ± 0.83** | 68.47 ± 1.12 | 68.44 ± 1.04 | 68.77 ± 1.01 | 68.70 ± 1.01 |
| Scala | NMI | 62.41 ± 0.67 | **62.76 ± 0.53** | 62.07 ± 0.68 | 62.28 ± 0.57 | 62.27 ± 0.80 | 62.18 ± 0.82 |
| Scala | ARI | 57.74 ± 1.09 | **58.59 ± 1.07** | 56.69 ± 1.59 | 57.13 ± 1.15 | 57.07 ± 1.68 | 56.91 ± 1.62 |
| Hrvatin B1 | ACC | 96.53 ± 0.56 | 96.41 ± 0.69 | 96.88 ± 0.59 | 96.72 ± 0.70 | **97.59 ± 0.09** | 97.53 ± 0.01 |
| Hrvatin B1 | NMI | 96.27 ± 0.47 | 96.22 ± 0.59 | 96.69 ± 0.63 | 96.57 ± 0.71 | **97.42 ± 0.12** | 97.20 ± 0.14 |
| Hrvatin B1 | ARI | 96.17 ± 0.20 | 96.32 ± 0.13 | 96.78 ± 0.53 | 96.52 ± 0.72 | **97.34 ± 0.14** | 97.12 ± 0.06 |
| PBMC 3K | ACC | 85.79 ± 0.05 | **86.03 ± 0.06** | 85.94 ± 0.13 | 85.86 ± 0.06 | 84.97 ± 0.36 | 84.23 ± 0.38 |
| PBMC 3K | NMI | 79.11 ± 0.01 | 79.31 ± 0.01 | 79.23 ± 0.07 | **79.35 ± 0.10** | 78.62 ± 0.47 | 78.26 ± 0.47 |
| PBMC 3K | ARI | 68.88± 0.04 | **69.39 ± 0.18** | 69.14 ± 0.23 | 69.06 ± 0.11 | 68.00 ± 0.38 | 67.50 ± 0.20 |
| Hrvatin B2 | ACC | 92.83 ± 0.02 | 93.09 ± 0.23 | **93.43 ± 0.07** | 93.03 ± 0.12 | 93.03 ± 0.12 | 92.85 ± 0.01 |
| Hrvatin B2 | NMI | 94.98 ± 0.01 | 93.23 ± 1.76 | 91.55 ± 0.06 | 93.18 ± 1.28 | 93.18 ± 1.28 | **94.99 ± 0.00** |
| Hrvatin B2 | ARI | 90.07 ± 0.03 | 88.95 ± 1.16 | 88.02 ± 0.16 | 88.87 ± 0.87 | 88.87 ± 0.87 | **90.09 ± 0.01** |
| Zhang | ACC | 90.90 ± 0.17 | 90.64 ± 0.39 | **91.24 ± 0.39** | 90.45 ± 0.05 | 90.56 ± 0.01 | 90.51 ± 0.25 |
| Zhang | NMI | 86.14 ± 0.22 | 85.74 ± 0.36 | 86.20 ± 0.10 | 86.50 ± 0.66 | **87.76 ± 0.14** | 85.96 ± 0.04 |
| Zhang | ARI | 90.26 ± 0.12 | 90.03 ± 0.41 | **90.27 ± 0.05** | 89.99 ± 0.04 | 90.13 ± 0.05 | 90.06 ± 0.05 |
| Savas | ACC | 76.04 ± 0.69 | **77.83 ± 0.43** | 75.99 ± 0.31 | 75.82 ± 0.34 | 74.77 ± 0.23 | 72.64 ± 0.46 |
| Savas | NMI | 60.15 ± 0.45 | **60.79 ± 0.47** | 59.45 ± 0.13 | 58.77 ± 0.15 | 59.36 ± 0.06 | 57.24 ± 0.54 |
| Savas | ARI | 61.84 ± 0.64 | **62.87 ± 0.37** | 60.77 ± 0.33 | 60.83 ± 0.57 | 59.92 ± 0.22 | 56.96 ± 0.15 |
